# Structural Foundations of Potassium Selectivity in Channelrhodopsins

**DOI:** 10.1128/mbio.03039-22

**Published:** 2022-11-22

**Authors:** Elena G. Govorunova, Oleg A. Sineshchekov, Leonid S. Brown, Ana-Nicoleta Bondar, John L. Spudich

**Affiliations:** a Center for Membrane Biology, Department of Biochemistry & Molecular Biology, The University of Texas Health Science Center at Houston McGovern Medical School, Houston, Texas, USA; b Department of Physics and Biophysics Interdepartmental Group, University of Guelph, Guelph, Ontario, Canada; c Faculty of Physics, University of Bucharest, Bucharest, Romania; d Institute of Computational Biomedicine, Forschungszentrum Jülich, Jülich, Germany; Columbia University

**Keywords:** channelrhodopsins, inhibition, optogenetics, patch clamp, potassium channels

## Abstract

Potassium-selective channelrhodopsins (KCRs) are light-gated K^+^ channels recently found in the stramenopile protist Hyphochytrium catenoides. When expressed in neurons, KCRs enable high-precision optical inhibition of spiking (optogenetic silencing). KCRs are capable of discriminating K^+^ from Na^+^ without the conventional K^+^ selectivity filter found in classical K^+^ channels. The genome of H. catenoides also encodes a third paralog that is more permeable for Na^+^ than for K^+^. To identify structural motifs responsible for the unusual K^+^ selectivity of KCRs, we systematically analyzed a series of chimeras and mutants of this protein. We found that mutations of three critical residues in the paralog convert its Na^+^-selective channel into a K^+^-selective one. Our characterization of homologous proteins from other protists (Colponema vietnamica, Cafeteria burkhardae, and Chromera velia) and metagenomic samples confirmed the importance of these residues for K^+^ selectivity. We also show that Trp102 and Asp116, conserved in all three *H. catenoides* paralogs, are necessary, although not sufficient, for K^+^ selectivity. Our results provide the foundation for further engineering of KCRs for optogenetic needs.

## INTRODUCTION

Channelrhodopsins (ChRs) are a diverse group of >500 light-gated ion channels found in eukaryotic microbes (1) and widely used as optogenetic tools (2). ChRs are members of a larger protein family known as microbial rhodopsins (3–5) and are composed of seven transmembrane helices (TM1 to TM7) with the retinal chromophore attached in a Schiff base linkage to a conserved Lys residue in the middle of TM7. In the model flagellate alga Chlamydomonas reinhardtii, the role of ChRs as phototaxis receptors has been established by analysis of knockdown genetic transformants (6). ChRs are thought to function similarly in other microorganisms, because all species in the genomes in which ChRs have been found produce flagellate gametes and/or zoospores during their life cycles. ChRs recently discovered in giant algal viruses are thought to mediate phototaxis of their hosts, thus enhancing host metabolism to support virus reproduction (7, 8).

By their ion selectivity, ChRs can be classified into three groups: anion-selective ChRs (ACRs), cation-selective ChRs (CCRs), and potassium channelrhodopsins (KCRs). ACRs conduct halides and nitrate, hyperpolarize the membrane in mature neurons, and inhibit their spiking (9). CCRs conduct primarily protons and, to a lesser extent, mono- and divalent metal cations (10). The relative permeability of CCRs for Na^+^ is greater than that for K^+^, so under physiological conditions they depolarize the membrane and activate neuronal spiking (11). Recently, we reported two potassium channelrhodopsins from the stramenopile fungus-like protist Hyphochytrium catenoides (*Hc*KCR1 and *Hc*KCR2) that are more permeable for K^+^ than Na^+^, and we demonstrated that these light-gated channels can be used to inhibit mouse cortical neurons (12). Notably, KCRs lack the K^+^ channel signature sequence universally found in K^+^ channels from bacteria, archaea, eukaryotic cells, and their viruses gated by voltage, ligands, heat, pH, or membrane deformation (13, 14). The ability of KCRs to discriminate between K^+^ and Na^+^ is particularly intriguing, because it reveals the only so far known alternative mechanism of K^+^ selectivity.

Cation conductance has appeared at least twice in microbial rhodopsin evolution, as CCRs from chlorophytes and streptophytes show very little protein sequence homology to CCRs from cryptophytes. Structurally and functionally, the latter resemble haloarchaeal proton-pumping rhodopsins such as bacteriorhodopsin, and they are therefore known as bacteriorhodopsin-like cation channelrhodopsins (BCCRs) (15). KCR protein sequences show the highest homology to cryptophyte BCCRs out of all currently known ChRs (12), although their source organism is phylogenetically very distant from cryptophytes. High-resolution structures of only one BCCR, known as ChRmine, have been reported (16, 17). They show trimeric organization typical of haloarchaeal ion-pumping rhodopsins (18), whereas chlorophyte CCRs and cryptophyte ACRs form dimers (19, 20).

In addition to two KCRs, the completely sequenced genome of *H. catenoides* encodes a third paralog (21). Surprisingly, this channel, named *H. catenoides* cation channelrhodopsin (*Hc*CCR), did not show higher permeability for K^+^ than for Na^+^ when tested by planar automated patch clamp (22). Thus, the three *H. catenoides* ChRs form a unique highly homologous group of light-gated channels with K^+^/Na^+^ permeability ratios differing over a wide range. In this study, we used them as a platform to elucidate the structural foundations of the K^+^ selectivity mechanism of KCRs. In addition, we tested 13 homologs from other protists and metagenomic samples and characterized those that are electrogenic. The results obtained confirmed our conclusions about the K^+^ selectivity mechanism drawn from analysis of *H. catenoides* ChRs.

## RESULTS

### Characterization of *Hc*CCR.

**FIG 1:**
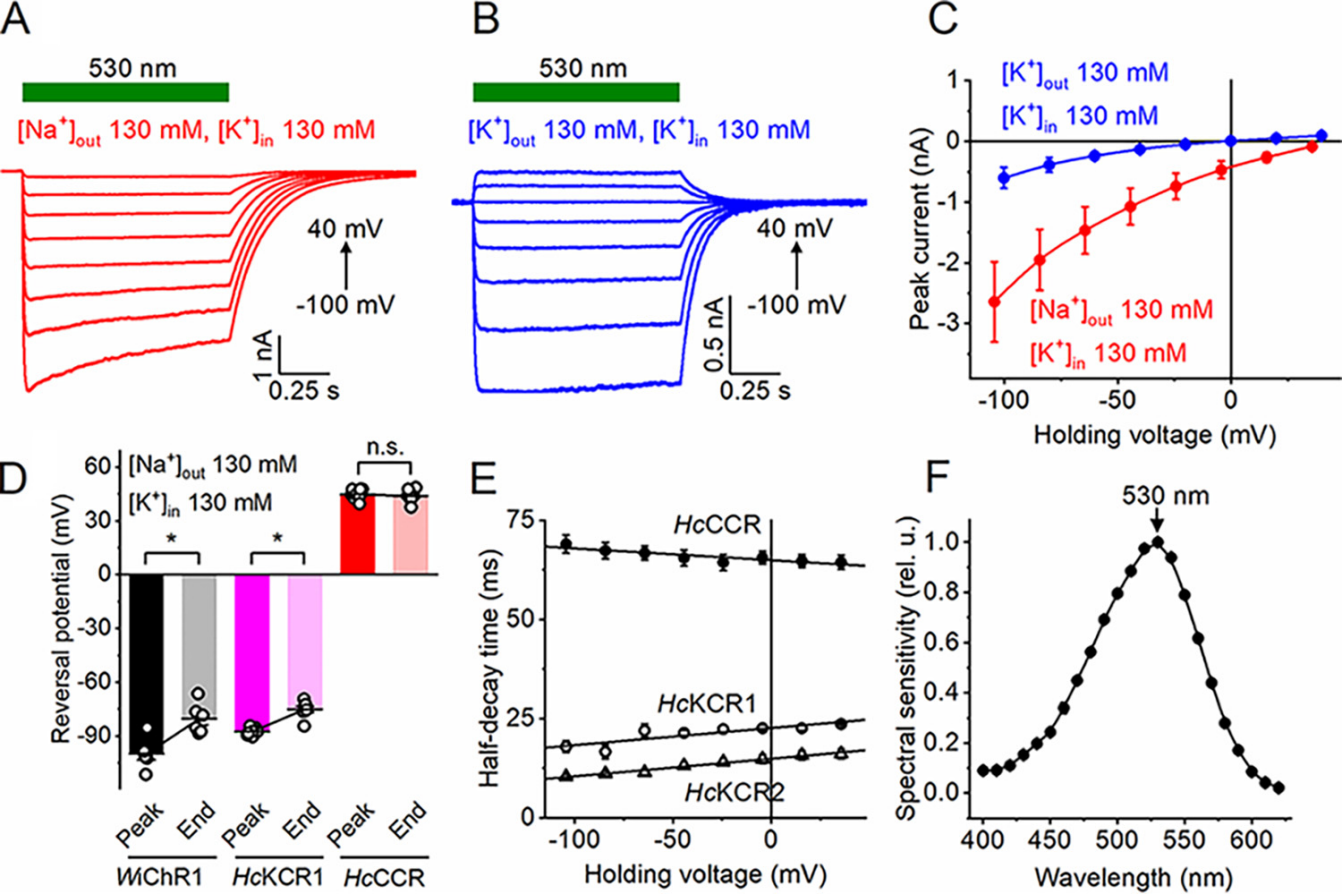
Electrophysiological characterization of *Hc*CCR. (A and B) Series of photocurrent traces recorded from *Hc*CCR upon incremental voltage with 130 mM Na^+^ (A) or K^+^ (B) in the bath and 130 mM K^+^ in the pipette. The duration of the light pulse is shown by the green bars. (C) The peak current-voltage relationships of *Hc*CCR under the indicated ionic conditions. The data points are the means ± SEMs (*n* = 7 and 6 cells for the Na^+^ and K^+^ bath, respectively). (D) The reversal potentials of the peak current and current at the end of 1-s illumination measured in the Na^+^ bath as in panel A. The data points are the means ± SEMs (*n* = 6 to 8 cells for each variant). *, *P* < 0.05 by the two-tailed paired sample Wilcoxon signed-rank test. n.s., not significant. The data for *Hc*KCR1 and *Hc*KCR2 are shown for comparison. (E) The dependence of the photocurrent half-decay time on the holding voltage for the three *H. catenoides* ChRs. The data points are the means ± SEMs (*n* = 6 cells for each variant); the lines are linear approximations. (F) Action spectrum of *Hc*CCR photocurrents. The data points are the means ± SEMs (*n* = 6 cells). The numerical data for panels C, D, E and F, including the exact numbers of cells sampled, are provided in Data Set S1, and full statistical analysis, including the exact *P* values, is in Data Set S2.

Previously, we tested *Hc*CCR expressed in HEK293 (human embryonic kidney) cells by automatic patch clamp using complex solutions that do not allow a straightforward estimation of the K^+^/Na^+^ permeability (P_K_/P_Na_) ratio (22). In this study, we measured its current-voltage relationship (IV curve) by manual patch clamp under bi-ionic conditions (130 mM NaCl in the bath and 130 mM KCl in the pipette; for full solution compositions, see Table S1 in the supplemental material), which we had used earlier to characterize *Hc*KCRs (12). Figure 1A shows a series of photocurrents generated by *Hc*CCR under incremental voltage, and Fig. 1C (red) shows the mean voltage dependence of the peak photocurrent. The reversal potential (V_rev_), estimated by approximation of the IV curve to zero current, was >40 mV under these conditions (Fig. 1C, red). Figure 1B shows a series of photocurrents generated by *Hc*CCR upon replacement of Na^+^ in the bath with K^+^. The mean IV curve measured with symmetrical K^+^ is shown in Fig. 1C (blue). The current amplitude was reduced, and the V_rev_ shifted to zero (Fig. 1C, blue). The P_K_/P_Na_ value, calculated from the V_rev_ shift using the Goldman-Hodgkin-Katz equation (23), was ~0.2. This value was 128-fold and 94-fold smaller than the P_K_/P_Na_ ratios of *Hc*KCR1 and *Hc*KCR2, respectively (12), and even smaller than that of ChR2 from C. reinhardtii, the best-characterized chlorophyte CCR (0.3 to 0.5 [10, 24]). Under bi-ionic conditions, *Hc*KCR1 showed a shift of V_rev_ to more depolarized values during 1-s illumination (12). This shift was even larger in the recently identified KCR from the stramenopile Wobblia lunata, named *Wobblia* inhibitory ChR1 (*W*iChR1) (Fig. 1D, black, and Fig. S1) (25). However, no such shift was detected in *Hc*CCR (Fig. 1D, red), which confirmed our previous conclusion that in *Hc*KCR1 this shift reflected a decrease in the P_K_/P_Na_ ratio during illumination (12). The decay of *Hc*CCR photocurrent slightly accelerated upon depolarization, but was slower than that in both *Hc*KCRs (Fig. 1E). The maximal spectral sensitivity of *Hc*CCR was at 530 nm (Fig. 1F).

10.1128/mbio.03039-22.3 FIG S1 Electrophysiological characterization of *W*iChR1. (A) Series of photocurrent traces recorded upon incremental voltage with 130 mM Na^+^ in the bath and 130 mM K^+^ in the pipette. The duration of the light pulse is shown by the green bars. (C) Peak current-voltage relationships of *W*iChR1. Black, mean ± SEM (*n* = 7 cells); gray, data from individual cells. For two cells in which the photocurrent at the positive voltages exceeded the dynamic range of the amplifier (20 nA), the peak values were obtained by fitting of a sigmoidal (dose-response) function to the data. The red arrow points to the reversal potential. Download FIG S1, TIF file, 0.2 MB. Copyright © 2022 Govorunova et al. 2022 Govorunova et al. https://creativecommons.org/licenses/by/4.0/ This content is distributed under the terms of the Creative Commons Attribution 4.0 International license.

10.1128/mbio.03039-22.9 TABLE S1 Solution compositions for whole-cell patch clamp recording. LJP, liquid junction potential. All concentrations are millimolar. Download Table S1, DOCX file, 0.01 MB. Copyright © 2022 Govorunova et al. 2022 Govorunova et al. https://creativecommons.org/licenses/by/4.0/ This content is distributed under the terms of the Creative Commons Attribution 4.0 International license.

**FIG 2:**
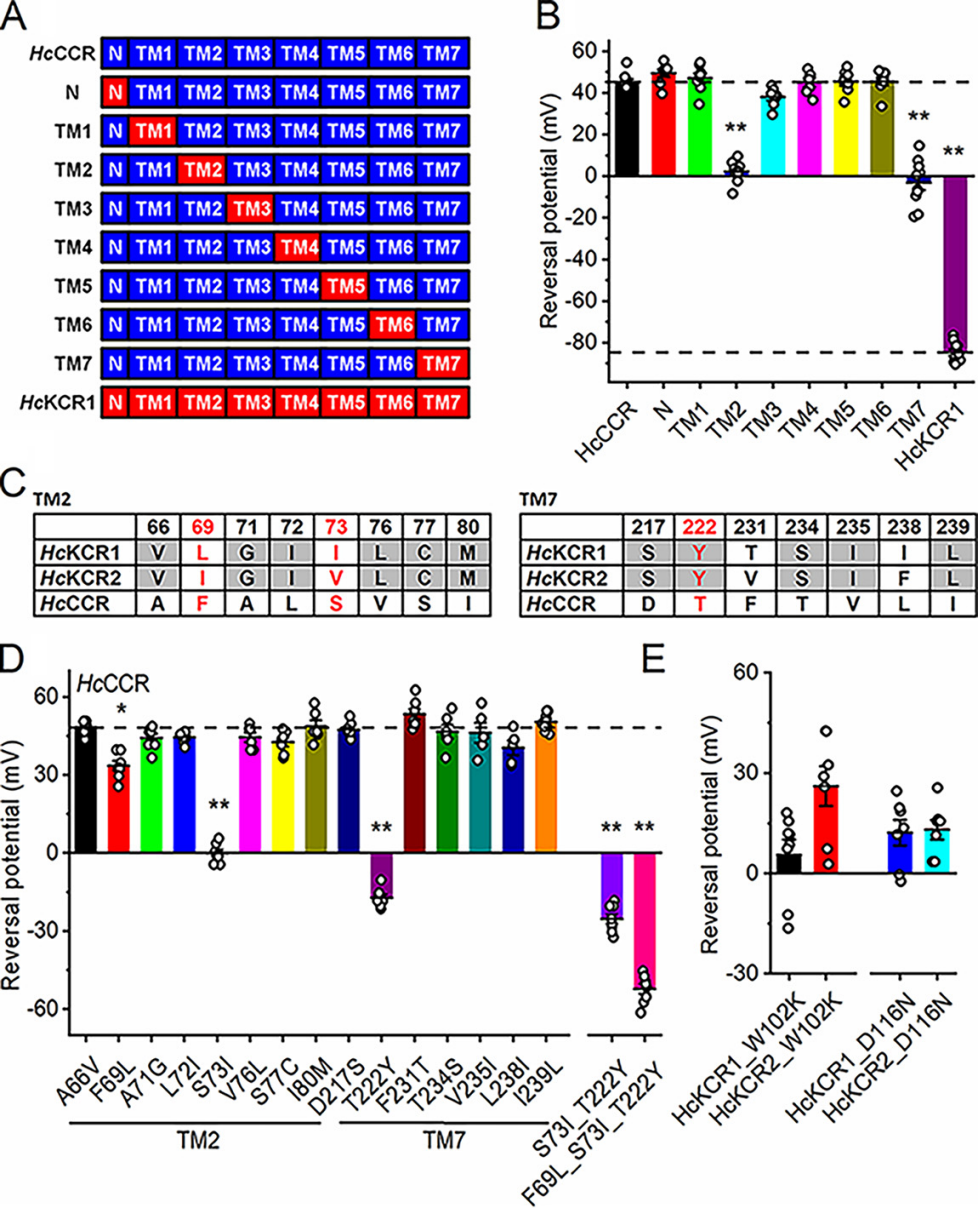
Analysis of *Hc*CCR chimeras and mutants. (A) Schematic representation of *Hc*KCR1_*Hc*CCR chimeras tested in this study. (B) V_rev_ values measured in the wild-type proteins and chimeras under bi-ionic conditions (130 mM Na^+^ in the bath and 130 mM K^+^ in the pipette). The dashed lines mark the V_rev_ value of wild-type *Hc*CCR and *Hc*KCR1. (C) The residues in the tested positions in TM2 and TM7 of the three *H. catenoides* ChRs. The red font shows the positions critical for K^+^ selectivity. (D) V_rev_ values measured in the single and multiple *Hc*CCR mutants as described for B. The dashed line marks the V_rev_ value of wild-type *Hc*CCR. (E) V_rev_ values measured in the W102K and D116N mutants of *Hc*KCR1 and *Hc*KCR2. In panels B, D and E, the bars and whiskers show the means ± SEMs (*n* = 5 to 10 cells); the empty circles show the data for individual cells. *, *P* < 0.05; **, *P* < 0.01 by one-way ANOVA followed by the Tukey test for means comparison. The numerical data for panels B, D, and E are provided in Data Set S1, and their full statistical analysis is in Data Set S2.

10.1128/mbio.03039-22.1 DATA SET S1 Numerical data shown in Fig. 1C to F, Fig. 2B, D, and E, and Fig. 4D to G in the main text. Download Data Set S1, XLSX file, 0.02 MB. Copyright © 2022 Govorunova et al. 2022 Govorunova et al. https://creativecommons.org/licenses/by/4.0/ This content is distributed under the terms of the Creative Commons Attribution 4.0 International license.

10.1128/mbio.03039-22.2 DATA SET S2 Full statistical analysis of the data shown in Fig. 1D and 2B and D and in the text. Download Data Set S2, PDF file, 0.2 MB. Copyright © 2022 Govorunova et al. 2022 Govorunova et al. https://creativecommons.org/licenses/by/4.0/ This content is distributed under the terms of the Creative Commons Attribution 4.0 International license.

### *Hc*CCR_*Hc*KCR1 chimeras and mutants.

The seven-transmembrane (7TM) domain of *Hc*CCR shares 70 to 73% identity and 83 to 86% similarity at the protein level with those of KCRs (Fig. S2A). Remarkably, the protein alignment shows no gaps, so the numbers of the homologous residues are the same in all three proteins. As the first step toward determination of the structural foundations of the K^+^ selectivity of *Hc*KCRs, we carried out patch clamp analysis of *Hc*CCR_*Hc*KCR1 chimeras. Starting with the *Hc*CCR sequence, we systematically replaced individual predicted helical regions with those of *Hc*KCR1. We have also created an additional chimera by replacement of the N-terminal region of *Hc*CCR with that of *Hc*KCR1. A protein alignment of the chimeras is shown in Fig. S2B, and their schematic representation is shown in Fig. 2A. Next, we measured the IV curves of the chimeras under bi-ionic conditions (Fig. S3A) and calculated the V_rev_ values as described above for wild-type *Hc*CCR. Remarkably, replacement of TM2 or TM7 caused a >40-mV shift of V_rev_ to more negative values, indicating a large increase in the P_K_/P_Na_ ratio (Fig. 2B). These results suggested that residues responsible for the K^+^ selectivity of *Hc*KCRs are located in TM2 and TM7.

10.1128/mbio.03039-22.4 FIG S2 (A) Protein alignment of 7TM domains of *H. catenoides* ChRs. The residues are shaded according to the degree of conservation. The numbers on the right are those of the last residue in each line. The red boxes show predicted transmembrane helices. (B) Protein alignment of the *Hc*KCR1_*Hc*CCR chimeras tested in this study. The residues are shaded according to the degree of conservation. The numbers on the right are those of the last residue in each line. Download FIG S2, TIF file, 2.3 MB. Copyright © 2022 Govorunova et al. 2022 Govorunova et al. https://creativecommons.org/licenses/by/4.0/ This content is distributed under the terms of the Creative Commons Attribution 4.0 International license.

10.1128/mbio.03039-22.5 FIG S3 (A) Peak current-voltage relationships of the *Hc*KCR1_*Hc*CCR chimeras. Black, mean ± SEM (*n* = 7 to 10 cells); gray, data from individual cells. The red arrows point to the reversal potentials. (B) Peak current-voltage relationships of the *Hc*CCR mutants. Black, mean ± SEM (*n* = 5 to 8 cells); gray, data from individual cells. The red arrows point to the reversal potentials. Download FIG S3, TIF file, 2.7 MB. Copyright © 2022 Govorunova et al. 2022 Govorunova et al. https://creativecommons.org/licenses/by/4.0/ This content is distributed under the terms of the Creative Commons Attribution 4.0 International license.

Next, we identified the positions in TM2 and TM7 occupied by the same residue in both *Hc*KCRs but not *Hc*CCR and the positions in which the residues are different in all three proteins (Fig. 2C). We individually replaced the residues of *Hc*CCR with those found in *Hc*KCR1 and measured the IV curves of the resultant point mutants under bi-ionic conditions (Fig. S3B). Only three mutations (F69L, S73I, and T222Y) caused a significant shift of V_rev_ toward more negative values (Fig. 2D), indicating that the mutated residue positions are critical for the K^+^ selectivity of *Hc*KCRs. The effect of the individual mutations was synergistic, as the single F69L mutation caused a larger V_rev_ when it was added to the *Hc*CCR_S73I_T222Y double mutant than when it was made in wild-type *Hc*CCR (Fig. 2D). The three mutations together (F69L, S73I, and T222Y) converted Na^+^-selective *Hc*CCR into a KCR with a P_K_/P_Na_ ratio of ~8. In *Hc*KCR2, which shows a slightly lower P_K_/P_Na_ ratio than *Hc*KCR1 (12), position 73 is occupied by Val instead of Ile. The *Hc*KCR2_V73I mutation caused a small but statistically significant shift of the V_rev_ from −74 ± 2 to −79 ± 1 mV (mean ± standard error of the mean [SEM], *n* = 7 and 8 cells for the wild type and the mutant, respectively; *P* ≤ 0.05 by the two-tailed Mann-Whitney test; for full statistical analysis, see Data Set S2), which corresponded to an increase of the P_K_/P_Na_ ratio from 17 to 22.

Arginine in position 82 of bacteriorhodopsin is a component of the complex counterion to the protonated retinylidene Schiff base (26) and is highly conserved in all microbial rhodopsins, including chlorophyte and streptophyte CCRs. As an exception, in BCCRs the prevalent residue in the corresponding position is Lys, and in all three H. catenoides paralogs the corresponding position is occupied by Trp102. The *Hc*KCR1_W102R mutation completely abolished photocurrents, whereas *Hc*KCR1_W102K did generate small currents (Fig. S4). Surprisingly, this mutant showed a small positive V_rev_ under our bi-ionic conditions (Fig. 2E, black), reflecting a dramatic decrease in the P_K_/P_Na_ ratio caused by the mutation. An even more positive V_rev_ was observed in the *Hc*KCR2_W102K mutant (Fig. 2E, red).

10.1128/mbio.03039-22.6 FIG S4 Characterization of Trp102 and Asp116 mutants of *Hc*KCR1 and *Hc*KCR2. (Left) Series of photocurrent traces recorded upon incremental voltage with 130 mM Na^+^ in the bath and 130 mM K^+^ in the pipette. The duration of the light pulse is shown by the green bars. (Right) Peak current-voltage relationships. Black, mean ± SEM (*n* = 7 or 8 cells); gray, data from individual cells. The red arrows point to the reversal potentials. Download FIG S4, TIF file, 0.4 MB. Copyright © 2022 Govorunova et al. 2022 Govorunova et al. https://creativecommons.org/licenses/by/4.0/ This content is distributed under the terms of the Creative Commons Attribution 4.0 International license.

The Asp residue in the Schiff base proton donor position (corresponding to Asp96 in bacteriorhodopsin) is conserved in all three *H. catenoides* paralogs, as in most cryptophyte BCCRs. Mutagenetic neutralization of this residue strongly inhibited photocurrents in both *Hc*KCRs (Fig. S4) and shifted the V_rev_ to more positive values (Fig. 2E, blue and cyan), indicating a decrease in the P_K_/P_Na_ ratio. We conclude that Trp102 and Asp116 are necessary, although not sufficient, for the K^+^ selectivity of *Hc*KCRs. The Asp116 mutations, but not the W102 mutations, also caused an inward rectification of the IV curves in both *Hc*KCRs (Fig. S4).

### Homology modeling of *Hc*KCR1 and *Hc*CCR.

**FIG 3:**
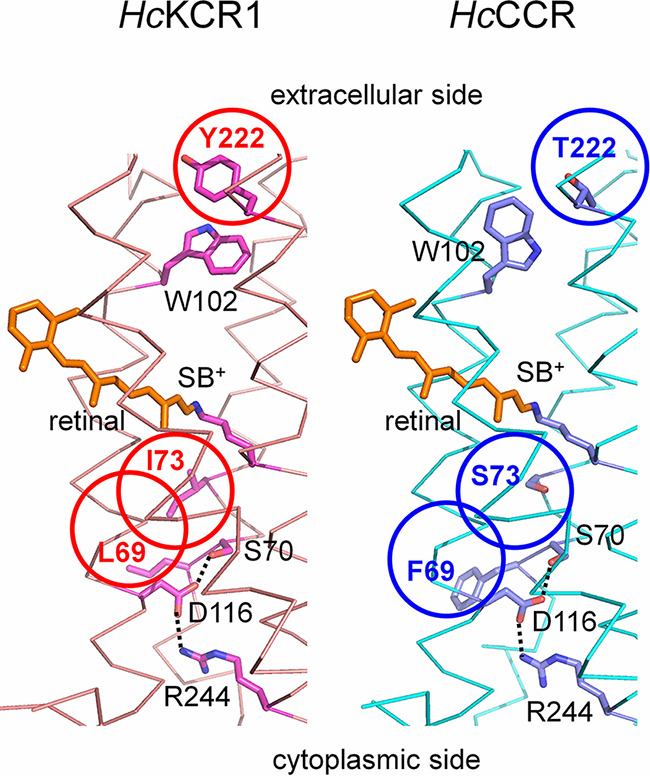
Homology models of *Hc*KCR1 (left) and *Hc*CCR (right). The side chains of the critical residues and the retinal chromophore are shown as sticks, the transmembrane helices as ribbons, and the predicted hydrogen bonds as dotted lines. The three residues responsible to the difference in the relative permeability are circled. SB^+^, protonated Schiff base.

To gain insight into locations of the critical residues identified in the previous section and predict their possible interactions, we created homology models of *Hc*KCR1 and *Hc*CCR (Fig. 3). The root mean square deviation (RMSD) of atomic positions between the two models is 0.7 Å. In both models, residues 69 and 73 are located in the cytoplasmic half of TM2 in the vicinity of Asp116, and residue 222 is near the extracellular surface of the protein within 5 Å of Trp102. All these residues are expected to contribute to the putative cation conduction pathway formed by TM1, -2, -3, and -7, as in other ChRs. In the *Hc*CCR model, the orientation of the Trp102 side chain is rotated upward from that in *Hc*KCR, likely as the result of the substitution of a more compact Thr for Tyr in position 222. This conformational difference, if confirmed by X-ray crystallography or cryo-electron microscopy (cryo-EM), may be relevant for control of the P_K_/P_Na_ ratio. Empirical calculations (27) predict that at pH 7.4 Asp116 is unprotonated in both channels (pK_a_ ~ 4). In both models, Asp116 forms side chain hydrogen bonds with Ser70 in the middle of TM2 and Arg 244 at the cytoplasmic end of TM7. In *Hc*KCR1, the S70A mutation decreased the P_K_/P_Na_ ratio (25), but Ser70 is conserved in *Hc*CCR and does not render this channel K^+^ selective. Considering the results of our mutant analysis (Fig. 2D), it is plausible that properties of Ser70 in *Hc*CCR compared to those in *Hc*KCR1 are modified by the substitutions of Phe for Leu and of Ser for Ile in the nearby positions 69 and 73, respectively. The latter, polar-to-nonpolar substitution produced a particularly large effect on the channel selectivity.

### KCR orthologs from other sources.

**FIG 4:**
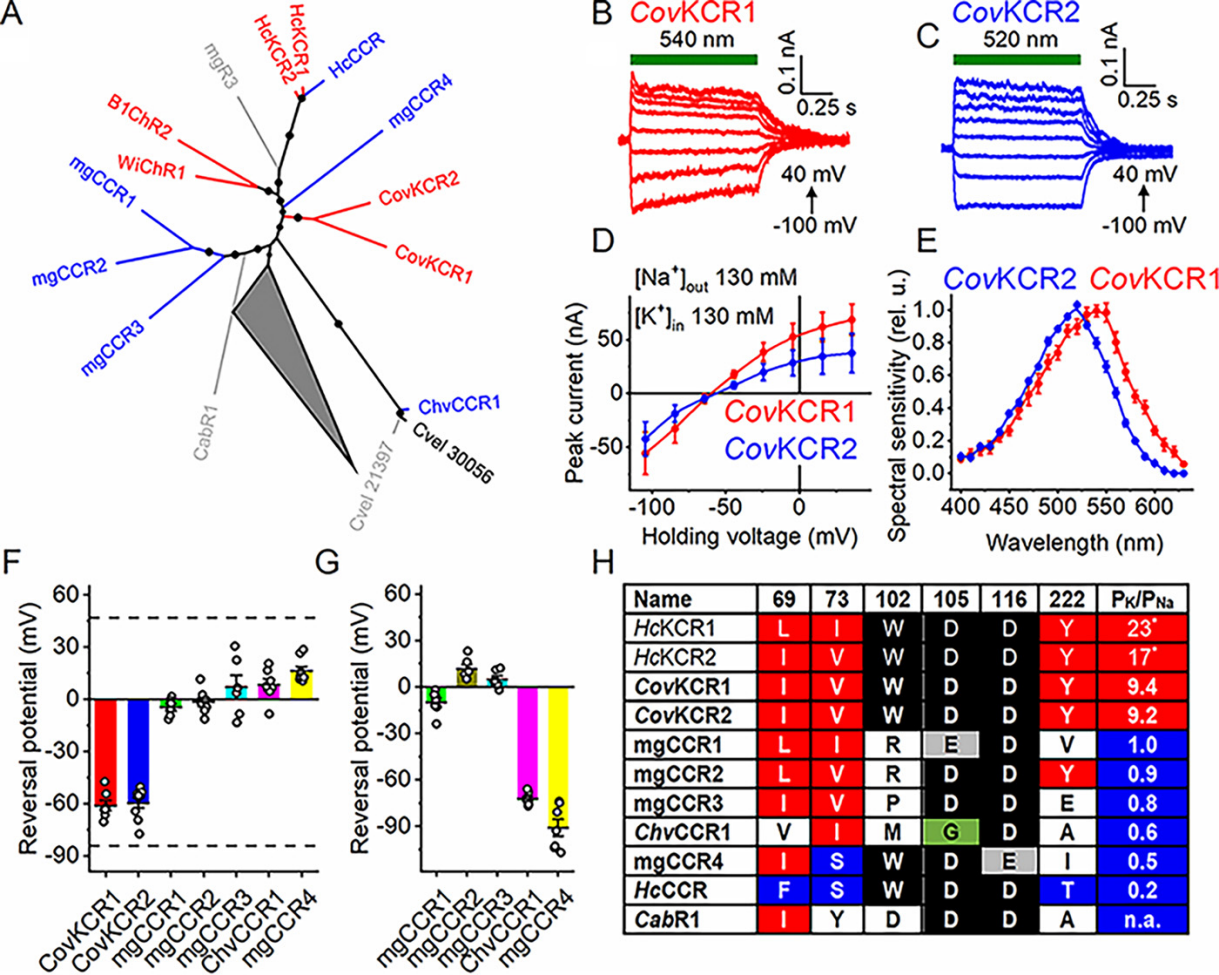
Characterization of KCR homologs. (A) Phylogenetic tree of the tested *Hc*KCR homologs. Variants that exhibit the K^+^ selectivity are in red, those that do not are in blue, and variants that do not generate channel currents are in gray. The collapsed node shows 510 other known ChRs (including nonfunctional mgR1 and mgR2). The 50 to 100 bootstrap values are shown as circles. (B and C) Series of photocurrent traces recorded from *Cov*KCRs upon incremental voltage with 130 mM Na^+^ in the bath and 130 mM K^+^ in the pipette. The green bars show the duration of the light pulse. (D) IV curves of *Cov*KCRs measured under these conditions. The data points are means ± SEMs (*n* = 7 and 9 cells for *Cov*KCR1 and *Cov*KCR2, respectively). (E) Action spectra of *Cov*KCR photocurrents. The data points are means ± SEMs (*n* = 7 cells for each variant). (F and G) V_rev_ values of the homologs measured under these conditions (F) and upon replacement of Na^+^ in the bath with NMDG^+^ (G). The bars and whiskers show the means ± SEMs (*n* = 6 to 9 cells); the empty circles show the data for individual cells. The dashed lines in panel F show the V_rev_ values for *Hc*KCR1 and *Hc*CCR from Fig. 2B. (H) Residues in the positions important for the K^+^ selectivity. Bold text shows variants tested in this study. *, value is from reference 12. n.a., not applicable. The red and blue backgrounds highlight the residues that, respectively, increase and decrease the K^+^ selectivity. The green background shows the noncarboxylate residue in the proton acceptor position in the C. velia sequence. The numerical data for panels D to G, including the exact numbers of cells sampled for each variant, are provided in Data Set S1.

To verify our conclusions about the structural foundations of the K^+^ selectivity drawn from the analysis of *Hc*CCR_*Hc*KCR1 chimeras and mutants, we searched the genomes and transcriptomes of other microorganisms and environmental samples for orthologs of *H. catenoides* ChRs. We identified 13 sequences that encode rhodopsin domains clustering together with *H. catenoides* ChRs on a phylogenetic tree (Fig. 4A). Among these, two sequences were found in the predatory alveolate Colponema vietnamica (28), one sequence was in the bicosoecid strain BVI (formerly considered Cafeteria roenbergensis but recently reattributed as Cafeteria burkhardae [Matthias Fischer, Max Planck Institute for Medical Research, personal communication]), three sequences were found in Chromera velia (an alga related to apicomplexan parasites [29]), and seven sequences were found in various metagenomic databases (listed in Materials and Methods). A protein alignment of their rhodopsin (7TM) domains is shown in Fig. S5. The three C. velia sequences and metagenomic MATOU-v2.32008995_3 and TARA_MED_95_MAG_00407_000000002956 differ from the rest by a noncarboxylate residue in the counterion position (corresponding to Asp85 in bacteriorhodopsin). A very unusual feature of the three C. velia sequences is substitution of Gly for Thr89 (bacteriorhodopsin numbering), which is nearly universally replaced with Cys in other known ChRs.

10.1128/mbio.03039-22.7 FIG S5 Protein alignment of 7TM domains of KCR homologs tested in this study. The residues are shaded according to the degree of conservation. In the C. velia sequences, the residues corresponding to Asp85 of bacteriorhodopsin are highlighted yellow, and the residues corresponding to Thr89 of bacteriorhodopsin are highlighted red. The numbers on the right are those of the last residue in each line. Download FIG S5, TIF file, 1.0 MB. Copyright © 2022 Govorunova et al. 2022 Govorunova et al. https://creativecommons.org/licenses/by/4.0/ This content is distributed under the terms of the Creative Commons Attribution 4.0 International license.

We synthesized mammalian codon-adapted versions of polynucleotides encoding these rhodopsins and expressed them as mCherry fusions in human embryonic kidney (HEK293) cells. Four homologs (Cvel_21397, MATOU-v2.141421879_4, MATOU-v2.32008995_3, and TARA_MED_95_MAG_00407_000000002956) were very poorly expressed and generated no photocurrents. We assigned the names metagenomic rhodopsins 1 to 3 (mgR1 to mgR3) to the sequences MATOU-v2.141421879_4, MATOU-v2.32008995_3, and TARA_MED_95_MAG_00407_000000002956, respectively. Cvel_30056 generated photocurrents, but they were smaller than those from the closely related Cvel_28437, so we did not characterize them. Photocurrents from Cvel_28437 (which we named *Chv*CCR1) and all other homologs except KAA0157615 from *C. burkhardae* demonstrated voltage-dependent sign reversal characteristic of passive conductance. We measured the IV curves under bi-ionic conditions, as described above for *Hc*CCR. Only two homologs (GILI01001652 and GILI01010992 from *C. vietnamica*) showed negative V_rev_ values, indicating their higher permeability for K^+^ over Na^+^. Figure 4B and C show series of their photocurrent traces, and Fig. 4D shows the corresponding IV curves. The action spectrum of GILI01001652 photocurrents peaked near 540 nm, and that of GILI01010992 peaked at 520 nm (Fig. 4E). Following the general ChR numbering convention, we named the more red-shifted paralog *Cov*KCR1 and the more blue-shifted one *Cov*KCR2. Representative photocurrent traces, the corresponding IV curves, and the spectra of other functional homologs are shown in Fig. S6A. All functional homologs showed inward rectification, also typical of many chlorophyte CCRs and cryptophyte BCCRs. We assigned the names mgCCR1 to mgCCR4 to the sequences Ga0170791_133102851, Ga0007756_110676931, MATOU-v2.119411731_5, and Ga0392354_009429_356_1807, respectively. When Na^+^ in the bath was replaced with nonpermeable *N*-methyl-d-gluconate (NMDG^+^), the IV curves for *Chv*CCR1 and mgCCR4 showed large shifts to the left, indicating a substantial permeability for Na^+^ (Fig. S6B). The IV curves for mgCCR1 to mgCCR3 showed little change, indicating that these homologs primarily conduct other ions, most probably H^+^. When the pH of the bath was raised to 9.4, the IV curves of all these three channels shifted to more negative voltages (Fig. S6C), which confirmed this hypothesis. The V_rev_ values measured in the Na^+^ and NMDG^+^ baths are shown in Fig. 4F and G, respectively.

10.1128/mbio.03039-22.8 FIG S6 (A) Characterization of KCR homologs with 130 mM Na^+^ in the bath. (Left) series of photocurrent traces recorded upon incremental voltage. The duration of the light pulse is shown by the colored bars. (Middle) Peak current-voltage relationships. Black, mean ± SEM (*n* = 6 to 9 cells); gray, data from individual cells. (Right) Action spectra of the photocurrents (mean ± SEM [*n* = 6 to 10 cells]). The red arrows point to the reversal potentials. (B) Characterization of KCR homologs with 130 mM NMDG^+^ in the bath. (Left) Series of photocurrent traces recorded upon incremental voltage. The duration of the light pulse is shown by the colored bars. (Right) Peak current-voltage relationships. Black, mean ± SEM (*n* = 6 or 7 cells); gray, data from individual cells. The red arrows point to the reversal potentials. (C) Characterization of KCR homologs with 130 mM K^+^ in the bath (pH 9.4). (Left) Series of photocurrent traces recorded upon incremental voltage. The duration of the light pulse is shown by the colored bars. (Right) Corresponding peak current-voltage relationships. The red arrows point to the reversal potentials. Download FIG S6, TIF file, 0.8 MB. Copyright © 2022 Govorunova et al. 2022 Govorunova et al. https://creativecommons.org/licenses/by/4.0/ This content is distributed under the terms of the Creative Commons Attribution 4.0 International license.

Figure 4H shows the residues in the critical positions in the sequences tested in this study and the earlier-characterized *Hc*KCRs. Only the K^+^-selective *C. vietnamica* sequences contain all residues identified as important for K^+^ selectivity in *Hc*KCRs, whereas in the non-K^+^-selective homologs some corresponding positions are occupied with nonhomologous residues. This strongly supports our conclusions about the structural determinants of K^+^ selectivity in ChRs. In particular, our results show that the presence of both the Trp102 homolog and the Tyr222 homolog is required for discrimination between K^+^ and Na^+^. Neither mgCCR4, in which only Trp102 is conserved but Tyr222 is replaced with Ile, nor mgCCR2, in which only Tyr222 is conserved but Trp102 is replaced with Arg, exhibit K^+^ selectivity.

Positive photocurrents from KAA0157615 recorded in the Na^+^ bath decayed with a time constant of ~15 ms after the onset of illumination and were practically independent of voltage (Fig. S6A). This behavior is typical of active intramolecular proton transfer from the Schiff base to an outwardly located acceptor (30). We assigned the name *Cafeteria burkhardae* rhodopsin 1 (*Cab*R1) to this protein.

## DISCUSSION

ChRs are found in many eukaryotic microbes, both photosynthetic and heterotrophic (1). During the last 17 years, ChRs have served as extremely powerful and versatile tools for optical control of the membrane potential in excitable cells such as neurons and myocytes, and they thus have become indispensable for neuroscience research (31). Moreover, partial recovery of visual function in a blind human patient by optogenetic means has launched a new era of ChR gene therapy (32). Our recent discovery of natural ChRs with a high P_K_/P_Na_ ratio (KCRs) (12) complements the inventory of optogenetic tools with long-sought, nearly universal inhibitory molecules.

Despite their prominence in biomedicine, the molecular mechanisms of ChRs, and especially the structural foundations of their ionic selectivity, are still poorly understood. Unlike conventional ion channels gated by voltage or ligands, in which the ion conductance pathway is formed at the interface between several subunits, it appears that each individual ChR protomer is capable of ion conductance. An interprotomer conductance has been proposed in ChRmine, based on the cryo-EM structure obtained in detergent (16). However, lipids block the space between the protomers in a ChRmine trimer incorporated in membranous nanodisks (17), which is expected to represent a state of the protein close to that in biological membranes.

In this study, we have taken advantage of the existence of closely homologous ChRs, *Hc*KCR1 and *Hc*CCR, that differ >100-fold in their P_K_/P_Na_ ratios. By systematic replacement of individual transmembrane helices of *Hc*CCR with those of *Hc*KCR1, we found that TM2 and TM7 are responsible for K^+^ selectivity. These helices contribute to the formation of the ion conduction pathway and channel gating in other ChRs, as shown by electron paramagnetic resonance (33, 34), electron crystallography (35), and X-ray crystallography (36). Then, we systematically mutated all divergent residues in TM2 and TM7 of *Hc*CCR, replacing them with those found in *Hc*KCR1 in the corresponding positions. We identified three residue positions, two in TM2 (69 and 73) and one in TM7 (222), critical for the K^+^ selectivity of the *Hc*KCRs. A lower P_K_/P_Na_ ratio of the triple mutant *Hc*CCR_F69L_S73I_T222Y than that of the wild-type *Hc*KCR1 is likely explained by synergy of other divergent residues that produce no significant effect individually but increase P_K_/P_Na_ in combination with other mutations, as we found for F69L. Phe/Leu69 and Ser/Ile73 are the homologs of Val49 and Ala53 of bacteriorhodopsin, respectively. In this protein, these residues control the position of the Schiff base lysine (Lys216) side chain relative to Asp85 (conserved in *H. catenoides* ChRs as Asp105) and affect distribution of the proton between them (37). In chlorophyte CCRs, the position of Ser/Ile73 is occupied by a highly conserved Glu residue (Glu90 in *Cr*ChR2). It contributes to the “central gate” and controls the selectivity of the channel; mutation of Glu90 to Lys or Arg renders *Cr*ChR2 permeable to anions (38). Considering the low protein sequence homology between *H. catenoides* ChRs and chlorophyte CCRs, the importance of this position in determination of the channel selectivity suggests a functional principle common to all ChRs.

Remarkably, two residues that are conserved in all three *H. catenoides* ChRs, namely, Trp102 and Asp116, are also required for the K^+^ selectivity of *Hc*KCRs, as we found by testing their W102K and D116N mutants. Trp102 corresponds to Arg82 of bacteriorhodopsin, which is highly conserved in microbial rhodopsins. Mutagenetic neutralization of the homologous residue (the R109N mutation) in the Na^+^-pumping rhodopsin from the flavobacterium Dokdonia eikasta (KR2) brings about weak passive K^+^ conductance (39). In cryptophyte BCCRs, the Arg82 position can be occupied by Pro, as in *Gt*CCR1 and *Gt*CCR2 from Guillardia theta (15), or even Glu, as in *Ra*CCR2 from Rhodomonas abbreviata (40), but not by Trp, as in KCRs.

Our homology model shows a close proximity of the Trp102 and Tyr222 positions in the extracellular portion of the putative cation pathway within *Hc*KCR1. In *W*iChR1 and a KCR from the stramenopile *Bilabrum* (*B1*ChR2 [25]), the residue corresponding to Tyr222 of *Hc*KCRs is Phe. Both of these KCRs showed higher P_K_/P_Na_ values than *Hc*KCR1, but the *Hc*KCR1_Y222F mutant exhibited a decrease rather than increase in K^+^ selectivity (25). Therefore, the Phe-for-Tyr substitution is unlikely responsible for the larger P_K_/P_Na_ ratio of *W*iChR1 and *B1*ChR2 than that of *Hc*KCR1. Several conserved aromatic residues are also found in the pore region of animal voltage-gated K^+^ channels, and the cation-π interaction has been proposed to contribute to their selectivity (41). A similar mechanism may be at work in microbial KCRs.

Asp116 of *Hc*KCRs corresponds to Asp96 of bacteriorhodopsin, the proton donor during reprotonation of the Schiff base (42). In chlorophyte CCRs, this Asp is replaced with a noncarboxylate residue (His173/His134 in *Cr*ChR1/*Cr*ChR2), and the *Cr*ChR1_H173D mutation completely abolished channel currents (43). In cryptophyte BCCRs, this Asp is conserved as in KCRs. In *Gt*CCR2 (one of the BCCRs), deprotonation of the Asp96 homolog (Asp98) occurs >10-fold faster than reprotonation of the Schiff base and is required for cation channel opening (15). While we were preparing our manuscript for submission, a preprint was published reporting patch clamp analysis of *Hc*KCR1 mutants (25). Its results provide an independent validation of the conclusions drawn in our study. In addition to Ser70, Trp102, and Asp116, described above, mutations of Asp87 and Asn99 also reduced the P_K_/P_Na_ ratio in *Hc*KCR1 (25), although both of these residues are conserved in Na^+^-selective *Hc*CCR. High-resolution structures are likely needed to explain this observation.

The P_K_/P_Na_ ratios of mammalian voltage-gated K^+^ channels fall within the range of 100 to 1,000 (14), which is higher than that of microbial KCRs. Our identification of the residues required for the K^+^ selectivity of ChRs is expected to facilitate both bioinformatic searches for potentially highly K^+^-selective ChR sequences and their molecular engineering to further improve the K^+^ selectivity. *Cov*KCR1 and *Cov*KCR2, tested in this study, are relatively poor candidates for the development of optogenetic tools, as their P_K_/P_Na_ ratios are lower than those of *Hc*KCRs and their photocurrents are very small. Nevertheless, these KCRs confirm the importance of the presence of both Trp102 and Tyr222 homologs for K^+^ selectivity. Also, their source organism, *C. vietnamica*, is phylogenetically very distant from *H. catenoides*, which suggests a wide distribution of KCRs in eukaryotic taxa. On the other hand, species attribution of transcripts derived from predatory microorganisms, such as *Colponema*, should be treated with caution, as there is a possibility of contamination of their transcriptomes with RNA from their food. This occurred, e.g., when ChRmine, the sequence encoded by the Cryptomonas lens genome, was erroneously attributed to the ciliate Tiarina fusus fed on *C. lens* (as discussed in reference 40).

Intramolecular proton transfers preceding channel currents were detected earlier in some chlorophyte CCRs (30) and cryptophyte BCCRs (15). Several chlorophyte sequences highly homologous to ChRs generate only fast photocurrents reflecting these transfers but show no passive ion conductance upon expression in mammalian cells (1), similar to *Cab*R1 described here. One possible explanation of the lack of channel activity in these proteins observed in heterologous systems is that they are more sensitive to membrane components, e.g., the lipid composition of the membrane, than other ChRs. (1). Only a small fraction of protist rhodopsins have been characterized at any level beyond their primary structure, and further characterization will likely bring many striking discoveries (44). Our results provide the foundation for further elucidating the K^+^ selection mechanism and for engineering KCRs for optogenetic applications.

## MATERIALS AND METHODS

### Bioinformatics and molecular biology.

*Hc*KCR homologs were identified by BLAST (BLASTP and TBLASTN) searches of various public databases, using a truncated TM domain amino acid sequence of *Hc*KCR1 (residues 13 to 251) as a query. Specifically, the GILI01001652 and GILI01010992 proteins of *C. vietnamica* strain Colp-7a were found using NCBI TBLASTN against the Transcriptome Shotgun Assembly (TSA) database limited to the SAR supergroup (taxid 2698737). The KAA0157615 protein of *Cafeteria burkhardae* strain BVI was found using NCBI BLASTP against the nonredundant (NR) protein database. The metagenomic MATOU-v2.141421879_4, MATOU-v2.32008995_3, MATOU-v2.119411731_5, and TARA_MED_95_MAG_00407_000000002956 proteins were found using BLASTP in the Ocean Gene Atlas (https://tara-oceans.mio.osupytheas.fr/ocean-gene-atlas/) (45, 46). MATOU-v2.141421879_4, MATOU-v2.32008995_3, and MATOU-v2.119411731_5 were extracted from the Marine Atlas of Tara Ocean Unigenes (MATOU [47]) data set, while TARA_MED_95_MAG_00407_000000002956 was found in Tara Oceans Single-Cell and Metagenome Assembled Genomes (EUK-SMAGs [48]) data set. The metagenomic Ga0392354_009429_356_1807, Ga0007756_110676931, and Ga0170791_133102851 proteins were found in the Department of Energy (DOE) Joint Genome Institute (JGI) Integrated Microbial Genomes and Microbiomes (IMG/M) database (49). BLASTP search was used against the respective metatranscriptomes (metatranscriptome of lab enriched marine microbial communities from Marineland, FL, USA, SWA_R2_TP1; metatranscriptome of freshwater lake microbial communities from Lake MI, USA, Su13.BD.MLB.DD; and Northern Canada Lakes metatranscriptome coassembly). Finally, amino acid sequences for Chromera velia proteins Cvel28437, Cvel21397, and Cvel30056 were found in PhycoCosm (https://phycocosm.jgi.doe.gov/Chrveli1/Chrveli1.home.html) (50) using BLASTP against the Chromera_velia 20200809 filtered protein model data set (51). Polynucleotides encoding the 7TM domains of the predicted proteins were optimized for mammalian expression. For expression in HEK293 (human embryonic kidney) cells, these polynucleotides and those encoding *H. catenoides* ChRs (GenBank accession numbers MZ826861, MZ826862, and OL692497) were cloned into the mammalian expression vector pcDNA3.1 (Life Technologies) in frame with a C-terminal mCherry tag.

The transmembrane helices were predicted using the DeepTMHMM algorithm (52). Sequences were aligned using MegAlign Pro software v. 17.1.1 (DNASTAR Lasergene) with default parameters. Phylogeny was analyzed with IQ-TREE v. 2.1.244 using automatic model selection and ultrafast bootstrap approximation (1,000 replicates) (53). iTOL v. 6.346 was used to visualize and annotate phylogenetic trees.

### Homology modeling of *Hc*KCR1 and *Hc*CCR.

We used ColabFold (54) with standard settings to generate, for each protein, five structural models based on multiple-sequence alignments. The predicted local distance difference test (pLDDT) confidence scores (54) of all models were in a relatively narrow range, 80.8 to 82.0 for *Hc*KCR1 and 81.8 to 83.4 for *Hc*CCR. A single structural model was chosen for each protein based on the overall similarity of the scores and the results of manual inspection for details of secondary structure and local interactions. We used the crystal structure of *Acetabularia* rhodopsin 1 (PDB code 5awz [55]) selected by ColabFold for model building to dock the retinal chromophore and internal water molecules to the ColabFold models by aligning them with the crystal structure of *Acetabularia* rhodopsin 1 in PyMol. We kept seven internal water molecules for *Hc*KCR1 and six for *Hc*CCR. Coordinates for missing hydrogen atoms were generated with CHARMM (Chemistry at HARvard Molecular Mechanics) (56). The retinal-bound models of *Hc*KCR1 and *Hc*CCR with internal water molecules were subjected to geometry optimizations using the CHARMM potential energy function with CHARMM36 parameters for water (57, 58), TIP3P water model (59), and retinal parameters as described previously (60–62). To optimize the geometry of retinal, water molecules, and protein groups within 3.5 Å of retinal and water, we fixed the coordinates of the heavy atoms of all other protein side chains: on the heavy atoms of the mobile groups, we initially placed harmonic constraints of 10 kcal mol^−1 ^Å^−2^ and energy optimized to a gradient of 0.1 kcal mol^−1 ^Å^−2^; we lowered the harmonic constraints first to 1.0 kcal mol^−1 ^Å^−2^ and then to 0.1 kcal mol^−1 ^Å^−2^, each time performing a new energy optimization to a gradient of 0.1 kcal mol^−1 ^Å^−2^. All harmonic constraints were then switched off and an additional energy optimization step was applied.

### Whole-cell patch clamp recording from HEK293 cells.

No cell lines from the list of known misidentified cell lines maintained by the International Cell Line Authentication Committee were used in this study. The HEK293 cells, from the American Type Culture Collection (ATCC), were grown in 2-cm-diameter plastic dishes and transfected with 10 μL of ScreenFectA transfection reagent (Waco Chemicals USA, Richmond, VA, USA) using 3 μg of DNA per dish. Immediately after transfection, all-*trans*-retinal (Sigma) was added at a final concentration of 5 μM. Patch pipettes with resistances of 2 to 3 MΩ were fabricated from borosilicate glass. Whole-cell voltage clamp recordings were performed with an Axopatch 200B amplifier (Molecular Devices) using the solutions, full composition of which is shown in Table S1, and a 4 M salt bridge. All measurements were carried out at room temperature (25°C). The signals were digitized with a Digidata 1440A controlled by pClampEx 10.7 software (both from Molecular Devices). All current-voltage curves (IV dependencies) were corrected for liquid junction potentials (LJP) calculated using the ClampEx built-in LJP calculator (Table S1). A Polychrome IV light source (T.I.L.L. Photonics GmbH) in combination with a mechanical shutter (Uniblitz model LS6; Vincent Associates; half-opening time, 0.5 ms) was used as the light source (maximal light power values at the focal plane of the 40× lens objective for all wavelengths used in this study are provided in Table S2). The action spectra were constructed using the initial slope of photocurrent in the linear range of the dependence on the quantum density (<25 μW mm^−2^), corrected for the quantum density measured at each wavelength and normalized to the maximal value. ClampFit 10.7 was used for initial analysis of the recorded data, followed by further analysis by Origin Pro 2016 software (OriginLab Corporation). The data points shown in the graphs are connected with spline or B-spline lines, unless otherwise stated.

10.1128/mbio.03039-22.10 TABLE S2 Maximal light power values for all excitation wavelengths used in this study. Download Table S2, DOCX file, 0.01 MB. Copyright © 2022 Govorunova et al. 2022 Govorunova et al. https://creativecommons.org/licenses/by/4.0/ This content is distributed under the terms of the Creative Commons Attribution 4.0 International license.

### Statistics and reproducibility.

Identical batches of HEK293 cell culture were randomly assigned for transfection with each tested construct. At least two separate batches of culture were transfected independently with each construct. Individual transfected cells were selected for patching by inspecting their tag fluorescence. Nonfluorescent cells or cells in which no GΩ seal could be established were not sampled. Only one photocurrent trace per cell was recorded, and traces recorded from different cells transfected with the same construct were considered biological replicates (reported as n values). Statistical analysis was performed using Origin Pro 2016 software. The data are presented as mean values ± SEMs; the data from individual cells are also shown when appropriate. Normal distribution of the data was checked using the Kolmogorov-Smirnov test. Specific statistical hypotheses were tested using the two-tailed paired-sample Wilcoxon signed-rank test (Fig. 1D), one-way analysis of variance (ANOVA) followed by the Tukey test for means comparison (Fig. 2B and D), and the two-tailed Mann-Whitney test (the data on the wild-type *Hc*KCR2 and its V73I mutant are in the text) as implemented in Origin. The complete results of hypothesis testing (including the exact numbers of cells tested for each variant and the exact *P* values) are provided in Data Set S4.

### Data availability.

The polynucleotide sequences of KCR homologs reported in this study have been deposited in GenBank (accession numbers OP121639 to OP121651). The numerical values of the data shown in Fig. 1, 2, and 4 are provided in Data Set S1.
