# Supplementary figures and images for: Structural Foundations of Potassium Selectivity in Channelrhodopsins
Source: mBio. 2022 Nov 22;13(6):e03039-22. doi: 10.1128/mbio.03039-22 (PMC9765531; doi:10.1128/mbio.03039-22)

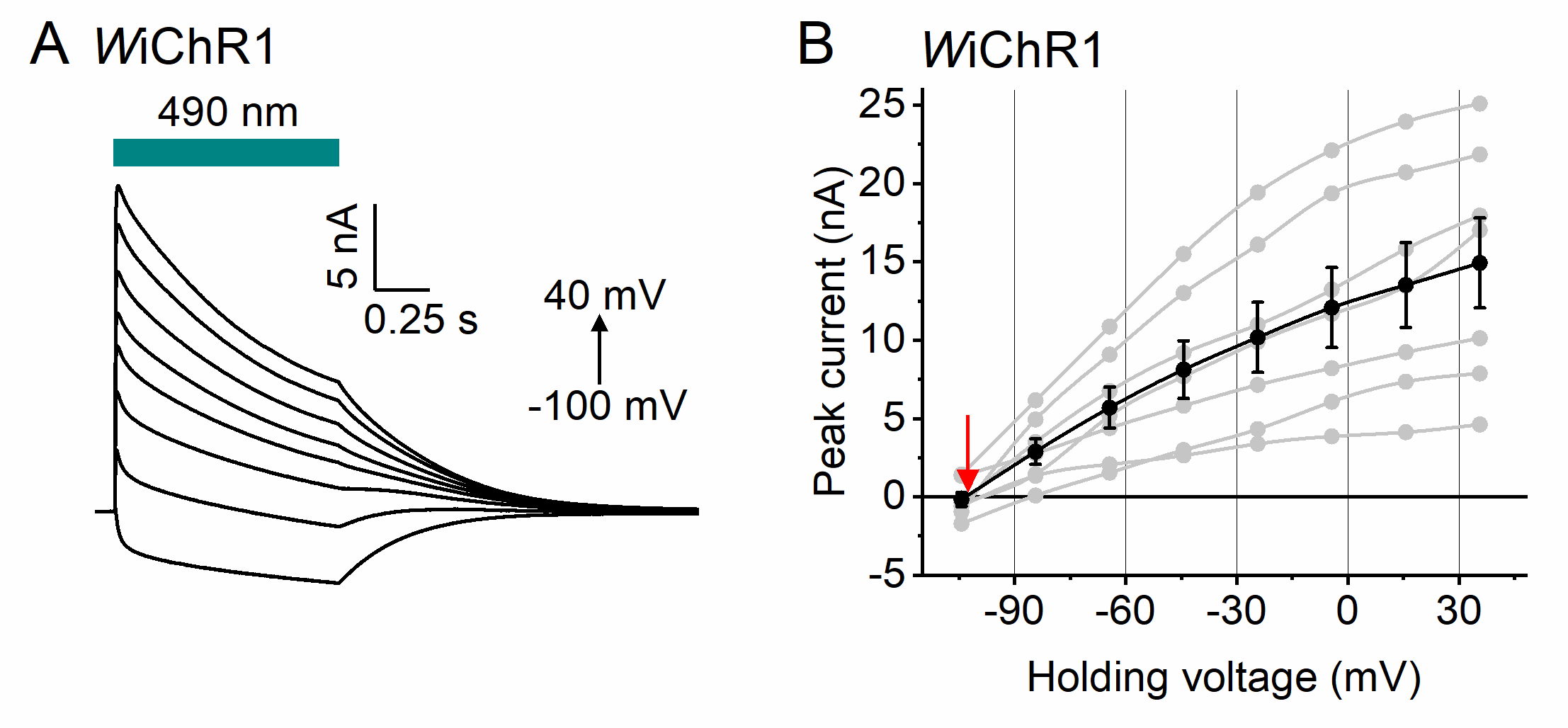

Supplement: FIG S1 [file mbio.03039-22-s0003.tif]

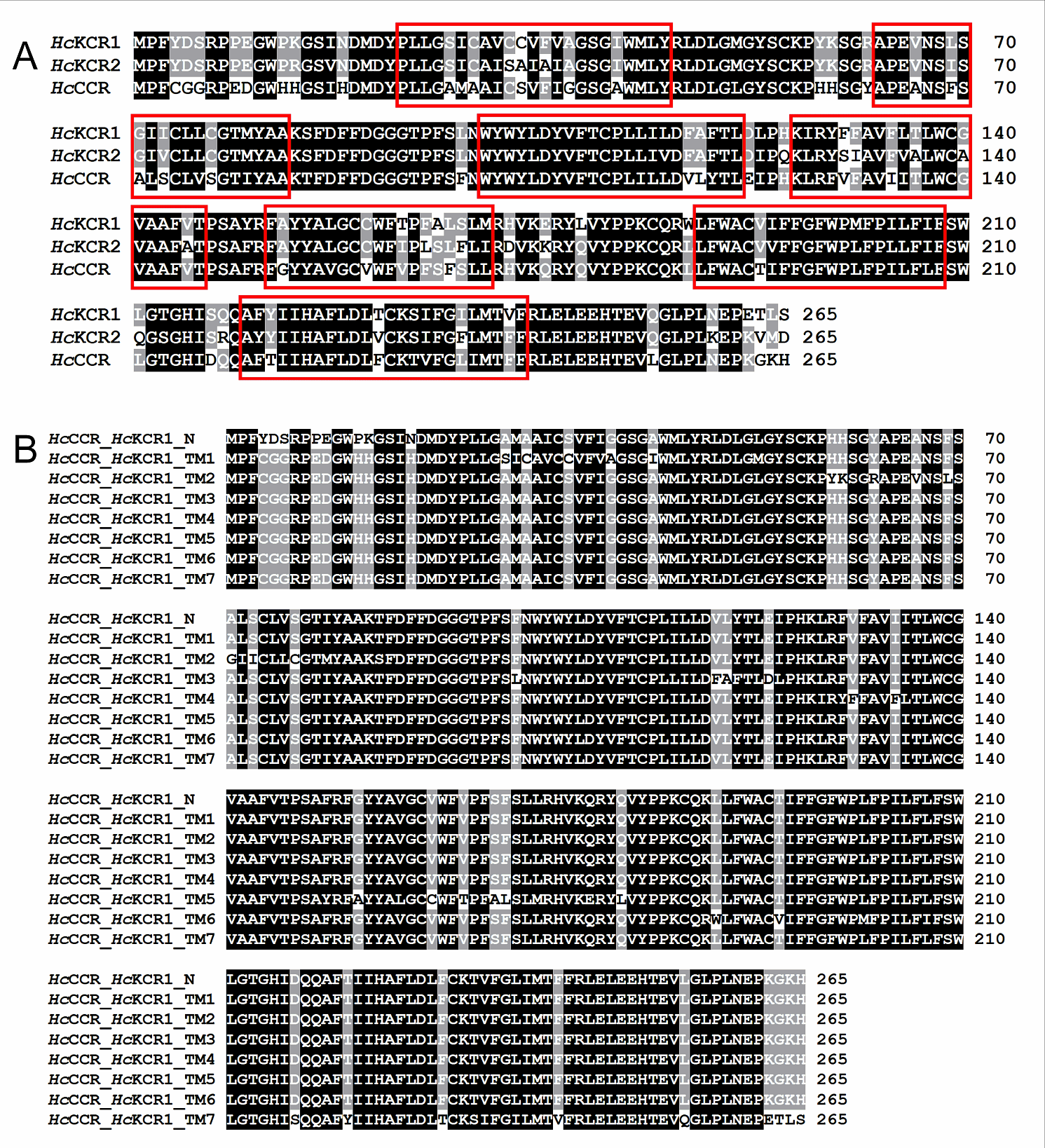

Supplement: FIG S2 [file mbio.03039-22-s0004.tif]

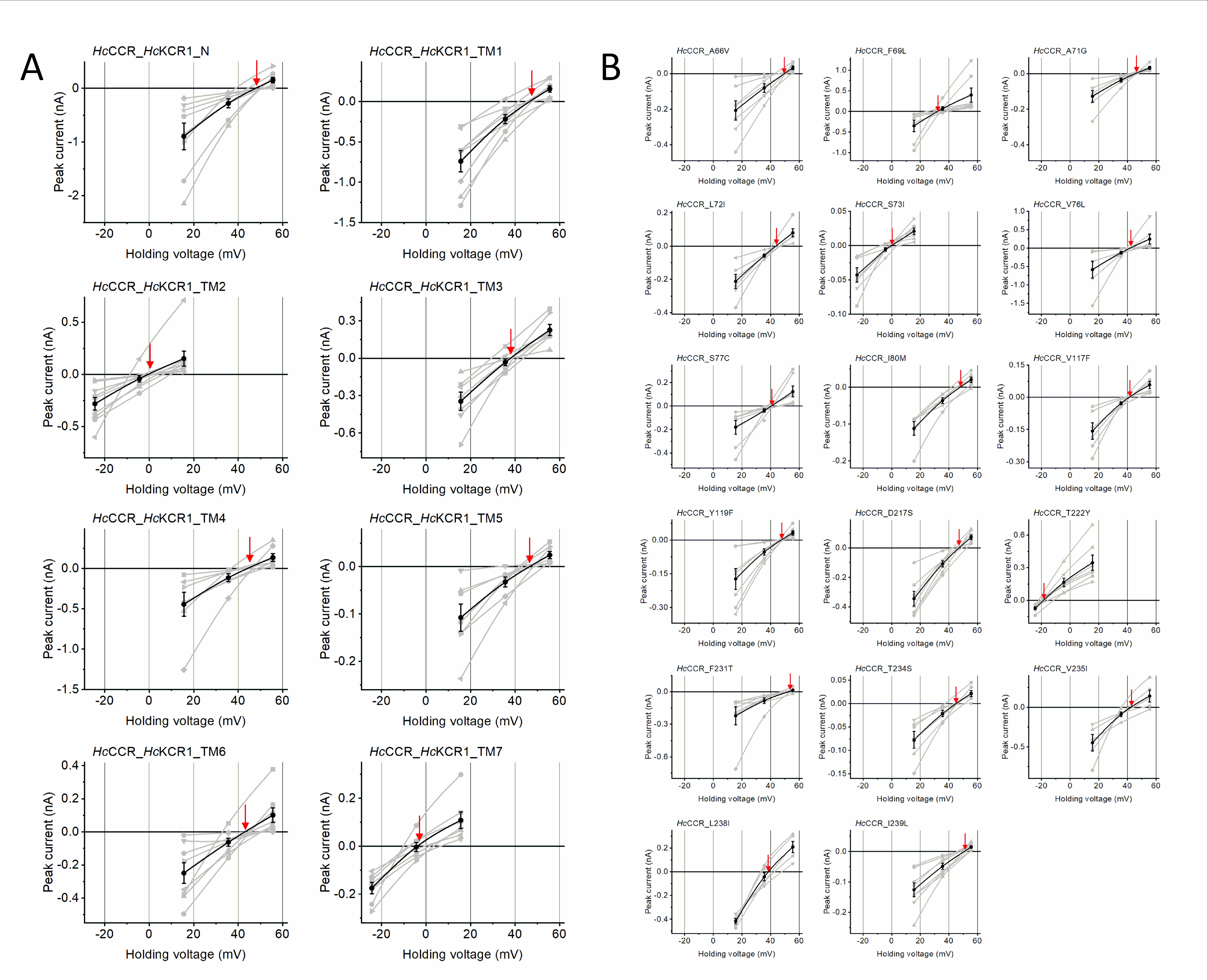

Supplement: FIG S3 [file mbio.03039-22-s0005.tif]

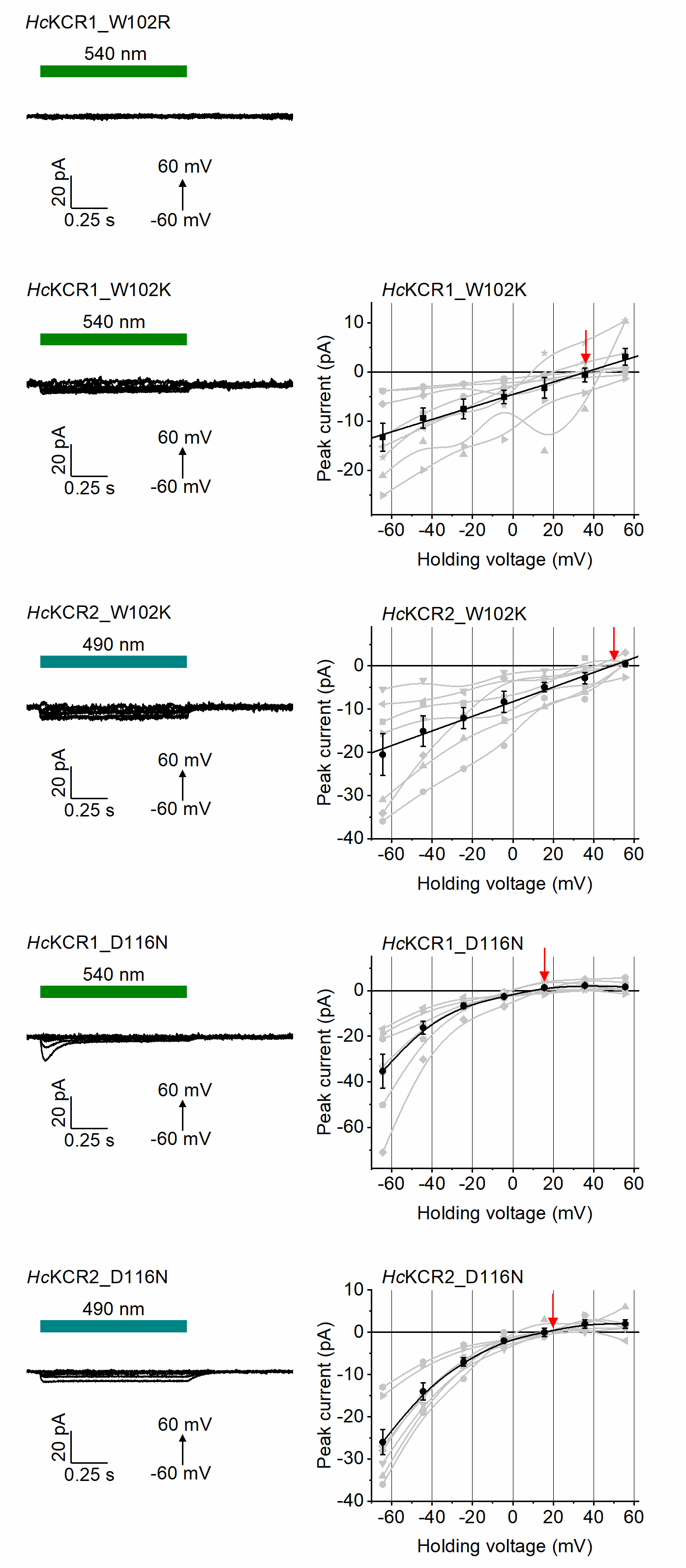

Supplement: FIG S4 [file mbio.03039-22-s0006.tif]

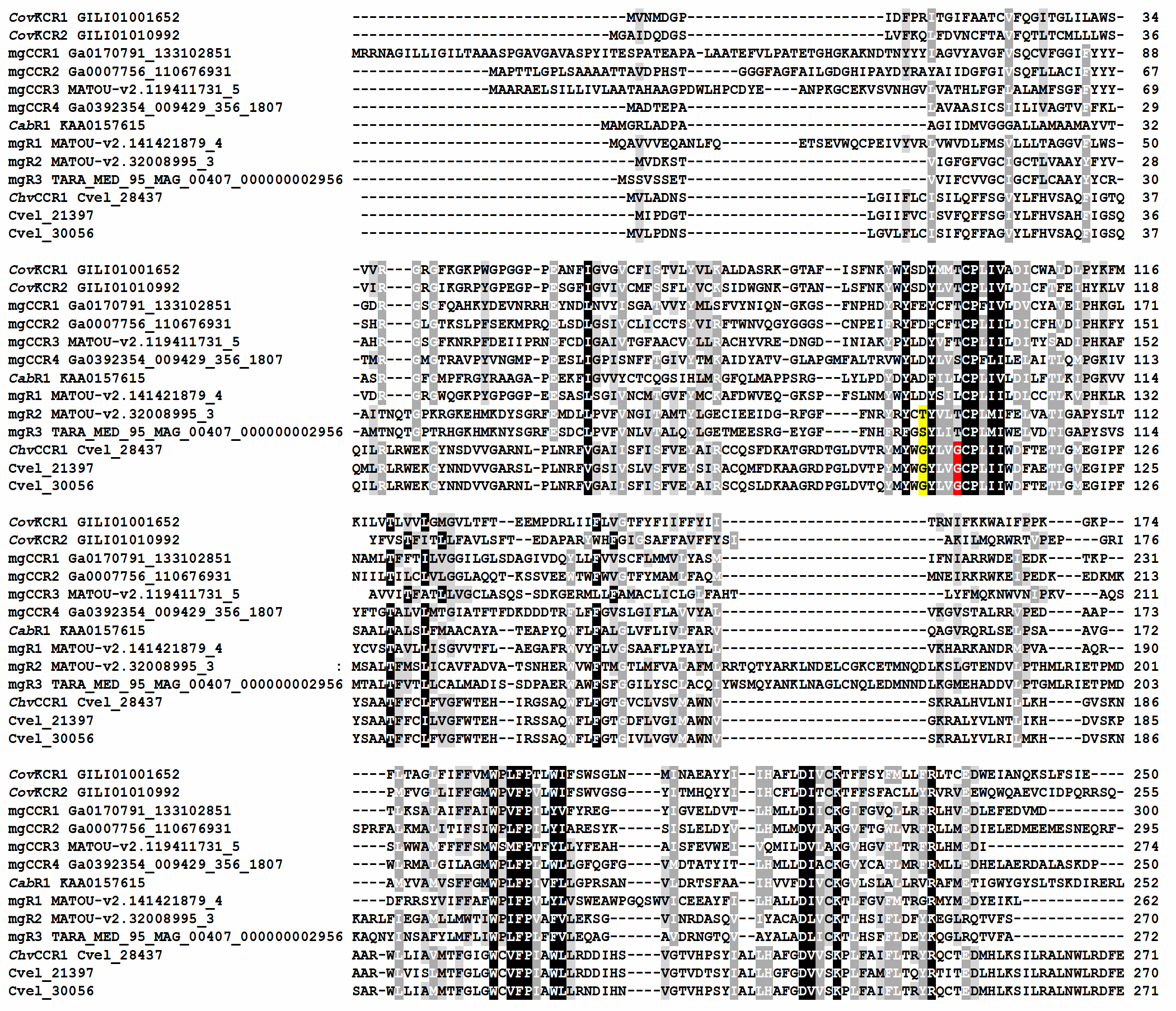

Supplement: FIG S5 [file mbio.03039-22-s0007.tif]
