# Supplementary material for: Structural Foundations of Potassium Selectivity in Channelrhodopsins
Source: mBio. 2022 Nov 22;13(6):e03039-22. doi: 10.1128/mbio.03039-22 (PMC9765531; doi:10.1128/mbio.03039-22)
Supplement: TABLE S1 [file mbio.03039-22-s0001.docx]

**Table S1.** Solution compositions for whole-cell patch clamp recording.

Abbreviations: HEPES, 4-(2-hydroxyethyl)-1-piperazineethanesulfonic acid; LJP, liquid junction potential. All concentrations are in mM.

|  | **NaCl** | **KCl** | **CaCl_2_** | **MgCl_2_** | **HEPES** | **Glucose** | **pH** | **LJP** |
| --- | --- | --- | --- | --- | --- | --- | --- | --- |
| **Pipette standard** | — | 130 | — | 2 | 10 | — | 7.4 | — |
| **Bath standard** | 130 | — | 2 | 2 | 10 | 10 | 7.4 | 4.4 |
| **Bath K^+^** | — | 130 | 2 | 2 | 10 | 10 | 7.4 | 0.2 |
| **Bath pH 9.4** | 150 | — | 2 | 2 | 10 | 10 | 9.4 | 4.4 |
