# Supplementary material for: Structural Foundations of Potassium Selectivity in Channelrhodopsins
Source: mBio. 2022 Nov 22;13(6):e03039-22. doi: 10.1128/mbio.03039-22 (PMC9765531; doi:10.1128/mbio.03039-22)
Supplement: DATA SET S2 [file mbio.03039-22-s0010.pdf]

Comparison of WiChR1 peak & end currents (Fig. 4D)

Wilcoxon Signed Ranks Test (9/21/2022 11:17:44)

Notes

X-Function	Wilcoxon Signed Ranks Test
User Name	egovorunova
Time	9/21/2022 11:17:44
Data Filter	No

Input Data

	Data	Range
1st Data Range	[DataErev]Sheet1!PeakWiChR1"Peak WiChR1"	[1*:6*]
2nd Data Range	[DataErev]Sheet1!EndWiChR1"End WiChR1"	[1*:6*]

Descriptive Statistics

	N	Min	Q1	Median	Q3	Max
"Peak WiChR1"	6	-111.4	-104.65	-100.9	-95.15	-85.4
"End WiChR1"	6	-88.4	-87.65	-81.9	-73.9	-66.4

Ranks

		N	Mean Rank	Sum Rank
"End WiChR1"-"Peak WiChR1"	Positive Ranks	6	3.5	21
	Negative Ranks	0	0	0

Test Statistics

	W	Z	Exact Prob>|W|	Asymp. Prob>|W|
	0	-2.09657	0.03125	0.03603

Null Hypothesis: F(x) = G(y)
Alternative Hypothesis: F(x) <> G(y)
At the 0.05 level, the two distributions are significantly different.

Comparison of HcKCR1 peak & end currents (Fig. 4D)

Wilcoxon Signed Ranks Test (9/21/2022 11:20:07)

Notes

X-Function	Wilcoxon Signed Ranks Test
User Name	egovorunova
Time	9/21/2022 11:20:07
Data Filter	No

Input Data

	Data	Range
1st Data Range	[DataErev]Sheet1!PeakHcKCR1"Peak HcKCR1"	[1*:7*]
2nd Data Range	[DataErev]Sheet1!EndHcKCR1"End HcKCR1"	[1*:7*]

Descriptive Statistics

	N	Min	Q1	Median	Q3	Max
"Peak HcKCR1"	7	-90.4	-89.4	-87.4	-85.4	-84.4
"End HcKCR1"	7	-84.4	-76.4	-74.4	-72.4	-69.4

Ranks

		N	Mean Rank	Sum Rank
"End HcKCR1"-"Peak HcKCR1"	Positive Ranks	7	4	28
	Negative Ranks	0	0	0

Test Statistics

	W	Z	Exact Prob>|W|	Asymp. Prob>|W|
	0	-2.30257	0.01563	0.0213

Null Hypothesis: F(x) = G(y)
Alternative Hypothesis: F(x) <> G(y)
At the 0.05 level, the two distributions are significantly different.

Comparison of HcCCR peak & end currents (Fig. 4D)

Wilcoxon Signed Ranks Test (9/21/2022 11:20:51)

Notes

X-Function	Wilcoxon Signed Ranks Test
User Name	egovorunova
Time	9/21/2022 11:20:51
Data Filter	No

Input Data

	Data	Range
1st Data Range	[DataErev]Sheet1!PeakHcCCR1"Peak HcCCR1"	[1*:8*]
2nd Data Range	[DataErev]Sheet1!EndHcCCR1"End HcCCR1"	[1*:8*]

Descriptive Statistics

	N	Min	Q1	Median	Q3	Max
"Peak HcCCR1"	8	39.6	42.85	44.6	47.6	47.6
"End HcCCR1"	8	37.6	42.6	43.6	46.85	48.6

Ranks

		N	Mean Rank	Sum Rank
"End HcCCR1"-"Peak HcCCR1"	Positive Ranks	2	2.5	5
	Negative Ranks	5	4.6	23

Test Statistics

	W	Z	Exact Prob>|W|	Asymp. Prob>|W|
	23	1.46584	0.17188	0.14269

Null Hypothesis: F(x) = G(y)

Alternative Hypothesis: F(x) <> G(y)

At the 0.05 level, the two distributions are NOT significantly different.

Comparison of HcCCR_HcKCR1 chimeras and HcCCR mutants (Fig. 2B, D)

ANOVAOneWay (8/4/2022 14:31:35)

Descriptive Statistics

	N Analysis	N Missing	Mean	Standard Deviation	SE of Mean
HcCCR	9	0	45.26667	4.06202	1.35401
N	8	0	49.475	4.99821	1.76714
TM1	8	0	47.1	6.39196	2.2599
TM2	9	0	2.15556	5.19882	1.73294
TM3	7	0	38.02857	4.64963	1.75739
TM4	7	0	45.02857	5.287	1.9983
TM5	7	0	45.45714	5.63999	2.13172
TM6	8	0	45.475	5.40998	1.91272
TM7	10	0	-3.2	11.01312	3.48266
A66V	7	0	48.31429	2.75162	1.04002
F69L	7	0	33.45714	5.08031	1.92018
A71G	7	0	44.17143	4.35343	1.64544
L72I	6	0	44.43333	2.40139	0.98036
S73I	7	0	-0.11429	3.77334	1.42619
V76L	6	0	44.43333	4.40076	1.7966
S77C	7	0	42.6	4.24264	1.60357
I80M	6	0	48.6	5.86515	2.39444
D217S	7	0	47.31429	3.03942	1.14879
T222Y	7	0	-17.25714	3.67099	1.3875
F231T	7	0	53.31429	5.2825	1.9966
T234S	6	0	46.6	6.57267	2.68328
V235I	5	0	46.2	8.59069	3.84187
L238I	5	0	40.4	6.30079	2.8178
I239L	8	0	50.35	3.41216	1.20638
S73I_T222Y	8	0	-25.4	5.15475	1.82248
F69L_S73I_T222	8	0	-52.275	5.38351	1.90336

One Way ANOVA

Overall ANOVA

	DF	Sum of Squares	Mean Square	F Value	Prob>F
Model	25	153975.12866	6159.00515	204.74729	9.48544E-109
Error	161	4843.04246	30.08101		
Total	186	158818.17112			

Null Hypothesis: The means of all levels are equal.
Alternative Hypothesis: The means of one or more levels are different.
At the 0.05 level, the population means are significantly different.

Fit Statistics

	R-Square	Coeff Var	Root MSE	Data Mean
	0.96951	0.18606	5.48462	29.47701

Means Comparisons

Tukey Test

	MeanDiff	SEM	q Value	Prob	Alpha	Sig	LCL	UCL
N HcCCR	4.20833	2.66504	2.23316	0.99786	0.05	0	-5.76916	14.18583
TM1 HcCCR	1.83333	2.66504	0.97286	1	0.05	0	-8.14416	11.81083
TM1 N	-2.375	2.74231	1.22479	1	0.05	0	-12.64176	7.89176
TM2 HcCCR	-43.11111	2.58547	23.58111	0	0.05	1	-52.79071	-33.43152
TM2 N	-47.31944	2.66504	25.1102	0	0.05	1	-57.29694	-37.34195
TM2 TM1	-44.94444	2.66504	23.8499	0	0.05	1	-54.92194	-34.96695
TM3 HcCCR	-7.2381	2.76399	3.70342	0.62266	0.05	0	-17.58602	3.10983
TM3 N	-11.44643	2.83856	5.70279	0.01935	0.05	1	-22.07354	-0.81932
TM3 TM1	-9.07143	2.83856	4.51953	0.22252	0.05	0	-19.69854	1.55568
TM3 TM2	35.87302	2.76399	18.35469	4.72423E-7	0.05	1	25.52509	46.22094
TM4 HcCCR	-0.2381	2.76399	0.12182	1	0.05	0	-10.58602	10.10983
TM4 N	-4.44643	2.83856	2.21528	0.9981	0.05	0	-15.07354	6.18068
TM4 TM1	-2.07143	2.83856	1.03202	1	0.05	0	-12.69854	8.55568
TM4 TM2	42.87302	2.76399	21.93629	0	0.05	1	32.52509	53.22094
TM4 TM3	7	2.93165	3.37677	0.78784	0.05	0	-3.97563	17.97563
TM5 HcCCR	0.19048	2.76399	0.09746	1	0.05	0	-10.15745	10.5384
TM5 N	-4.01786	2.83856	2.00176	0.99961	0.05	0	-14.64496	6.60925
TM5 TM1	-1.64286	2.83856	0.8185	1	0.05	0	-12.26996	8.98425
TM5 TM2	43.30159	2.76399	22.15557	0	0.05	1	32.95366	53.64951
TM5 TM3	7.42857	2.93165	3.58351	0.68682	0.05	0	-3.54706	18.4042
TM5 TM4	0.42857	2.93165	0.20674	1	0.05	0	-10.54706	11.4042
TM6 HcCCR	0.20833	2.66504	0.11055	1	0.05	0	-9.76916	10.18583
TM6 N	-4	2.74231	2.06281	0.99937	0.05	0	-14.26676	6.26676
TM6 TM1	-1.625	2.74231	0.83802	1	0.05	0	-11.89176	8.64176
TM6 TM2	43.31944	2.66504	22.98759	0	0.05	1	33.34195	53.29694
TM6 TM3	7.44643	2.83856	3.70993	0.61911	0.05	0	-3.18068	18.07354
TM6 TM4	0.44643	2.83856	0.22242	1	0.05	0	-10.18068	11.07354
TM6 TM5	0.01786	2.83856	0.0089	1	0.05	0	-10.60925	10.64496
TM7 HcCCR	-48.46667	2.52001	27.19922	0	0.05	1	-57.90117	-39.03216
TM7 N	-52.675	2.60158	28.634	0	0.05	1	-62.4149	-42.9351
TM7 TM1	-50.3	2.60158	27.34296	0	0.05	1	-60.0399	-40.5601
TM7 TM2	-5.35556	2.52001	3.00551	0.91921	0.05	0	-14.79006	4.07895
TM7 TM3	-41.22857	2.70285	21.57206	0	0.05	1	-51.3476	-31.10954
TM7 TM4	-48.22857	2.70285	25.23468	0	0.05	1	-58.3476	-38.10954
TM7 TM5	-48.65714	2.70285	25.45892	0	0.05	1	-58.77617	-38.53811
TM7 TM6	-48.675	2.60158	26.45961	0	0.05	1	-58.4149	-38.9351
A66V HcCC	3.04762	2.76399	1.55934	1	0.05	0	-7.3003	13.39554
A66V N	-1.16071	2.83856	0.57829	1	0.05	0	-11.78782	9.46639
A66V TM1	1.21429	2.83856	0.60498	1	0.05	0	-9.41282	11.84139
A66V TM2	46.15873	2.76399	23.61745	0	0.05	1	35.81081	56.50665
A66V TM3	10.28571	2.93165	4.96178	0.10019	0.05	0	-0.68991	21.26134
A66V TM4	3.28571	2.93165	1.58501	0.99999	0.05	0	-7.68991	14.26134
A66V TM5	2.85714	2.93165	1.37827	1	0.05	0	-8.11849	13.83277
A66V TM6	2.83929	2.83856	1.41458	1	0.05	0	-7.78782	13.46639
A66V TM7	51.51429	2.70285	26.95386	0	0.05	1	41.39526	61.63332
F69L HcCCR	-11.80952	2.76399	6.04243	0.00817	0.05	1	-22.15745	-1.4616
F69L N	-16.01786	2.83856	7.98034	2.2742E-5	0.05	1	-26.64496	-5.39075
F69L TM1	-13.64286	2.83856	6.79708	9.85542E-4	0.05	1	-24.26996	-3.01575
F69L TM2	31.30159	2.76399	16.01568	0	0.05	1	20.95366	41.64951
F69L TM3	-4.57143	2.93165	2.20523	0.99822	0.05	0	-15.54706	6.4042
F69L TM4	-11.57143	2.93165	5.582	0.02589	0.05	1	-22.54706	-0.5958
F69L TM5	-12	2.93165	5.78874	0.01565	0.05	1	-22.97563	-1.02437
F69L TM6	-12.01786	2.83856	5.98748	0.00944	0.05	1	-22.64496	-1.39075
F69L TM7	36.65714	2.70285	19.18015	1.5008E-7	0.05	1	26.53811	46.77617
F69L A66V	-14.85714	2.93165	7.16701	3.20034E-4	0.05	1	-25.83277	-3.88151
A71G HcCC	-1.09524	2.76399	0.56039	1	0.05	0	-11.44316	9.25268
A71G N	-5.30357	2.83856	2.64232	0.9796	0.05	0	-15.93068	5.32354
A71G TM1	-2.92857	2.83856	1.45906	1	0.05	0	-13.55568	7.69854
A71G TM2	42.01587	2.76399	21.49772	0	0.05	1	31.66795	52.3638
A71G TM3	6.14286	2.93165	2.96328	0.92946	0.05	0	-4.83277	17.11849
A71G TM4	-0.85714	2.93165	0.41348	1	0.05	0	-11.83277	10.11849
A71G TM5	-1.28571	2.93165	0.62022	1	0.05	0	-12.26134	9.68991
A71G TM6	-1.30357	2.83856	0.64946	1	0.05	0	-11.93068	9.32354
A71G TM7	47.37143	2.70285	24.78619	0	0.05	1	37.2524	57.49046
A71G A66V	-4.14286	2.93165	1.99849	0.99962	0.05	0	-15.11849	6.83277
A71G F69L	10.71429	2.93165	5.16852	0.06564	0.05	0	-0.26134	21.68991
L72I HcCCR	-0.83333	2.89065	0.4077	1	0.05	0	-11.65545	9.98878
L72I N	-5.04167	2.96203	2.40713	0.99378	0.05	0	-16.13104	6.0477
L72I TM1	-2.66667	2.96203	1.27319	1	0.05	0	-13.75604	8.4227
L72I TM2	42.27778	2.89065	20.68389	0	0.05	1	31.45566	53.09989
L72I TM3	6.40476	3.05136	2.96842	0.92826	0.05	0	-5.01904	17.82856
L72I TM4	-0.59524	3.05136	0.27588	1	0.05	0	-12.01904	10.82856
L72I TM5	-1.02381	3.05136	0.47451	1	0.05	0	-12.44761	10.39999
L72I TM6	-1.04167	2.96203	0.49734	1	0.05	0	-12.13104	10.0477
L72I TM7	47.63333	2.83224	23.78458	0	0.05	1	37.02987	58.2368
L72I A66V	-3.88095	3.05136	1.79871	0.99994	0.05	0	-15.30475	7.54284
L72I F69L	10.97619	3.05136	5.08714	0.0778	0.05	0	-0.44761	22.39999
L72I A71G	0.2619	3.05136	0.12139	1	0.05	0	-11.16189	11.6857
S73I HcCCR	-45.38095	2.76399	23.21949	0	0.05	1	-55.72887	-35.03303
S73I N	-49.58929	2.83856	24.70615	0	0.05	1	-60.21639	-38.96218
S73I TM1	-47.21429	2.83856	23.52289	0	0.05	1	-57.84139	-36.58718
S73I TM2	-2.26984	2.76399	1.16138	1	0.05	0	-12.61776	8.07808
S73I TM3	-38.14286	2.93165	18.39992	4.75842E-7	0.05	1	-49.11849	-27.16723
S73I TM4	-45.14286	2.93165	21.77669	0	0.05	1	-56.11849	-34.16723
S73I TM5	-45.57143	2.93165	21.98343	0	0.05	1	-56.54706	-34.5958
S73I TM6	-45.58929	2.83856	22.71329	0	0.05	1	-56.21639	-34.96218
S73I TM7	3.08571	2.70285	1.61454	0.99999	0.05	0	-7.03332	13.20474
S73I A66V	-48.42857	2.93165	23.3617	0	0.05	1	-59.4042	-37.45294
S73I F69L	-33.57143	2.93165	16.19469	0	0.05	1	-44.54706	-22.5958
S73I A71G	-44.28571	2.93165	21.36321	0	0.05	1	-55.26134	-33.31009
S73I L72I	-44.54762	3.05136	20.64649	0	0.05	1	-55.97142	-33.12382
V76L HcCCR	-0.83333	2.89065	0.4077	1	0.05	0	-11.65545	9.98878
V76L N	-5.04167	2.96203	2.40713	0.99378	0.05	0	-16.13104	6.0477
V76L TM1	-2.66667	2.96203	1.27319	1	0.05	0	-13.75604	8.4227
V76L TM2	42.27778	2.89065	20.68389	0	0.05	1	31.45566	53.09989
V76L TM3	6.40476	3.05136	2.96842	0.92826	0.05	0	-5.01904	17.82856
V76L TM4	-0.59524	3.05136	0.27588	1	0.05	0	-12.01904	10.82856
V76L TM5	-1.02381	3.05136	0.47451	1	0.05	0	-12.44761	10.39999
V76L TM6	-1.04167	2.96203	0.49734	1	0.05	0	-12.13104	10.0477
V76L TM7	47.63333	2.83224	23.78458	0	0.05	1	37.02987	58.2368
V76L A66V	-3.88095	3.05136	1.79871	0.99994	0.05	0	-15.30475	7.54284
V76L F69L	10.97619	3.05136	5.08714	0.0778	0.05	0	-0.44761	22.39999
V76L A71G	0.2619	3.05136	0.12139	1	0.05	0	-11.16189	11.6857
V76L L72I	-7.10543E	3.16654	3.17336E	1	0.05	0	-11.85503	11.85503
V76L S73I	44.54762	3.05136	20.64649	0	0.05	1	33.12382	55.97142
S77C HcCC	-2.66667	2.76399	1.36442	1	0.05	0	-13.01459	7.68126
S77C N	-6.875	2.83856	3.42523	0.7656	0.05	0	-17.50211	3.75211
S77C TM1	-4.5	2.83856	2.24197	0.99773	0.05	0	-15.12711	6.12711
S77C TM2	40.44444	2.76399	20.69369	0	0.05	1	30.09652	50.79237
S77C TM3	4.57143	2.93165	2.20523	0.99822	0.05	0	-6.4042	15.54706
S77C TM4	-2.42857	2.93165	1.17153	1	0.05	0	-13.4042	8.54706
S77C TM5	-2.85714	2.93165	1.37827	1	0.05	0	-13.83277	8.11849
S77C TM6	-2.875	2.83856	1.43237	1	0.05	0	-13.50211	7.75211
S77C TM7	45.8	2.70285	23.96397	0	0.05	1	35.68097	55.91903
S77C A66V	-5.71429	2.93165	2.75654	0.9668	0.05	0	-16.68991	5.26134
S77C F69L	9.14286	2.93165	4.41047	0.26441	0.05	0	-1.83277	20.11849
S77C A71G	-1.57143	2.93165	0.75805	1	0.05	0	-12.54706	9.4042
S77C L72I	-1.83333	3.05136	0.8497	1	0.05	0	-13.25713	9.59046
S77C S73I	42.71429	2.93165	20.60516	0	0.05	1	31.73866	53.68991
S77C V76L	-1.83333	3.05136	0.8497	1	0.05	0	-13.25713	9.59046
I80M HcCCR	3.33333	2.89065	1.63079	0.99999	0.05	0	-7.48878	14.15545
I80M N	-0.875	2.96203	0.41777	1	0.05	0	-11.96437	10.21437
I80M TM1	1.5	2.96203	0.71617	1	0.05	0	-9.58937	12.58937
I80M TM2	46.44444	2.89065	22.72238	0	0.05	1	35.62233	57.26656
I80M TM3	10.57143	3.05136	4.89954	0.11311	0.05	0	-0.85237	21.99523
I80M TM4	3.57143	3.05136	1.65525	0.99999	0.05	0	-7.85237	14.99523
I80M TM5	3.14286	3.05136	1.45662	1	0.05	0	-8.28094	14.56665
I80M TM6	3.125	2.96203	1.49202	1	0.05	0	-7.96437	14.21437
I80M TM7	51.8	2.83224	25.8651	0	0.05	1	41.19653	62.40347
I80M A66V	0.28571	3.05136	0.13242	1	0.05	0	-11.13808	11.70951
I80M F69L	15.14286	3.05136	7.01826	5.06251E-4	0.05	1	3.71906	26.56665
I80M A71G	4.42857	3.05136	2.05251	0.99942	0.05	0	-6.99523	15.85237
I80M L72I	4.16667	3.16654	1.86088	0.99989	0.05	0	-7.68837	16.0217
I80M S73I	48.71429	3.05136	22.57762	0	0.05	1	37.29049	60.13808
I80M V76L	4.16667	3.16654	1.86088	0.99989	0.05	0	-7.68837	16.0217
I80M S77C	6	3.05136	2.78082	0.96342	0.05	0	-5.4238	17.4238
D217S HcCC	2.04762	2.76399	1.04768	1	0.05	0	-8.3003	12.39554
D217S N	-2.16071	2.83856	1.0765	1	0.05	0	-12.78782	8.46639
D217S TM1	0.21429	2.83856	0.10676	1	0.05	0	-10.41282	10.84139
D217S TM2	45.15873	2.76399	23.10579	0	0.05	1	34.81081	55.50665
D217S TM3	9.28571	2.93165	4.47938	0.23739	0.05	0	-1.68991	20.26134
D217S TM4	2.28571	2.93165	1.10262	1	0.05	0	-8.68991	13.26134
D217S TM5	1.85714	2.93165	0.89588	1	0.05	0	-9.11849	12.83277
D217S TM6	1.83929	2.83856	0.91636	1	0.05	0	-8.78782	12.46639
D217S TM7	50.51429	2.70285	26.43063	0	0.05	1	40.39526	60.63332
D217S A66V	-1	2.93165	0.4824	1	0.05	0	-11.97563	9.97563
D217S F69L	13.85714	2.93165	6.68462	0.00137	0.05	1	2.88151	24.83277
D217S A71G	3.14286	2.93165	1.5161	1	0.05	0	-7.83277	14.11849
D217S L72I	2.88095	3.05136	1.33524	1	0.05	0	-8.54284	14.30475
D217S S73I	47.42857	2.93165	22.87931	0	0.05	1	36.45294	58.4042
D217S V76L	2.88095	3.05136	1.33524	1	0.05	0	-8.54284	14.30475
D217S S77C	4.71429	2.93165	2.27415	0.9972	0.05	0	-6.26134	15.68991
D217S I80M	-1.28571	3.05136	0.59589	1	0.05	0	-12.70951	10.13808
T222Y HcCC	-62.52381	2.76399	31.99076	0	0.05	1	-72.87173	-52.17589
T222Y N	-66.73214	2.83856	33.24698	0	0.05	1	-77.35925	-56.10504
T222Y TM1	-64.35714	2.83856	32.06372	0	0.05	1	-74.98425	-53.73004
T222Y TM2	-19.4127	2.76399	9.93265	4.21357E-8	0.05	1	-29.76062	-9.06478
T222Y TM3	-55.28571	2.93165	26.66955	0	0.05	1	-66.26134	-44.31009
T222Y TM4	-62.28571	2.93165	30.04632	0	0.05	1	-73.26134	-51.31009
T222Y TM5	-62.71429	2.93165	30.25306	0	0.05	1	-73.68991	-51.73866
T222Y TM6	-62.73214	2.83856	31.25412	0	0.05	1	-73.35925	-52.10504
T222Y TM7	-14.05714	2.70285	7.35513	1.7711E-4	0.05	1	-24.17617	-3.93811
T222Y A66V	-65.57143	2.93165	31.63133	0	0.05	1	-76.54706	-54.5958
T222Y F69L	-50.71429	2.93165	24.46432	0	0.05	1	-61.68991	-39.73866
T222Y A71G	-61.42857	2.93165	29.63284	0	0.05	1	-72.4042	-50.45294
T222Y L72I	-61.69048	3.05136	28.59169	0	0.05	1	-73.11427	-50.26668
T222Y S73I	-17.14286	2.93165	8.26963	8.44153E-6	0.05	1	-28.11849	-6.16723
T222Y V76L	-61.69048	3.05136	28.59169	0	0.05	1	-73.11427	-50.26668
T222Y S77C	-59.85714	2.93165	28.87479	0	0.05	1	-70.83277	-48.88151
T222Y I80M	-65.85714	3.05136	30.52282	0	0.05	1	-77.28094	-54.43335
T222Y D217	-64.57143	2.93165	31.14894	0	0.05	1	-75.54706	-53.5958
F231T HcCC	8.04762	2.76399	4.11762	0.39897	0.05	0	-2.3003	18.39554
F231T N	3.83929	2.83856	1.91279	0.99982	0.05	0	-6.78782	14.46639
F231T TM1	6.21429	2.83856	3.09605	0.89401	0.05	0	-4.41282	16.84139
F231T TM2	51.15873	2.76399	26.17573	0	0.05	1	40.81081	61.50665
F231T TM3	15.28571	2.93165	7.37375	1.66921E-4	0.05	1	4.31009	26.26134
F231T TM4	8.28571	2.93165	3.99699	0.46194	0.05	0	-2.68991	19.26134
F231T TM5	7.85714	2.93165	3.79025	0.57499	0.05	0	-3.11849	18.83277
F231T TM6	7.83929	2.83856	3.90565	0.51143	0.05	0	-2.78782	18.46639
F231T TM7	56.51429	2.70285	29.57002	0	0.05	1	46.39526	66.63332
F231T A66V	5	2.93165	2.41198	0.99361	0.05	0	-5.97563	15.97563
F231T F69L	19.85714	2.93165	9.57899	9.59938E-8	0.05	1	8.88151	30.83277
F231T A71G	9.14286	2.93165	4.41047	0.26441	0.05	0	-1.83277	20.11849
F231T L72I	8.88095	3.05136	4.11606	0.39976	0.05	0	-2.54284	20.30475
F231T S73I	53.42857	2.93165	25.77368	0	0.05	1	42.45294	64.4042
F231T V76L	8.88095	3.05136	4.11606	0.39976	0.05	0	-2.54284	20.30475
F231T S77C	10.71429	2.93165	5.16852	0.06564	0.05	0	-0.26134	21.68991
F231T I80M	4.71429	3.05136	2.18493	0.99845	0.05	0	-6.70951	16.13808
F231T D217	6	2.93165	2.89437	0.94418	0.05	0	-4.97563	16.97563
F231T T222	70.57143	2.93165	34.04331	0	0.05	1	59.5958	81.54706
T234S HcCC	1.33333	2.89065	0.65232	1	0.05	0	-9.48878	12.15545
T234S N	-2.875	2.96203	1.37266	1	0.05	0	-13.96437	8.21437
T234S TM1	-0.5	2.96203	0.23872	1	0.05	0	-11.58937	10.58937
T234S TM2	44.44444	2.89065	21.7439	0	0.05	1	33.62233	55.26656
T234S TM3	8.57143	3.05136	3.9726	0.47504	0.05	0	-2.85237	19.99523
T234S TM4	1.57143	3.05136	0.72831	1	0.05	0	-9.85237	12.99523
T234S TM5	1.14286	3.05136	0.52968	1	0.05	0	-10.28094	12.56665
T234S TM6	1.125	2.96203	0.53713	1	0.05	0	-9.96437	12.21437
T234S TM7	49.8	2.83224	24.86645	0	0.05	1	39.19653	60.40347
T234S A66V	-1.71429	3.05136	0.79452	1	0.05	0	-13.13808	9.70951
T234S F69L	13.14286	3.05136	6.09132	0.00718	0.05	1	1.71906	24.56665
T234S A71G	2.42857	3.05136	1.12557	1	0.05	0	-8.99523	13.85237
T234S L72I	2.16667	3.16654	0.96766	1	0.05	0	-9.68837	14.0217
T234S S73I	46.71429	3.05136	21.65068	0	0.05	1	35.29049	58.13808
T234S V76L	2.16667	3.16654	0.96766	1	0.05	0	-9.68837	14.0217
T234S S77C	4	3.05136	1.85388	0.99989	0.05	0	-7.4238	15.4238
T234S I80M	-2	3.16654	0.89322	1	0.05	0	-13.85503	9.85503
T234S D217	-0.71429	3.05136	0.33105	1	0.05	0	-12.13808	10.70951
T234S T222	63.85714	3.05136	29.59588	0	0.05	1	52.43335	75.28094
T234S F231	-6.71429	3.05136	3.11187	0.88914	0.05	0	-18.13808	4.70951
V235I HcCC	0.93333	3.05917	0.43147	1	0.05	0	-10.51972	12.38638
V235I N	-3.275	3.12671	1.48128	1	0.05	0	-14.98091	8.43091
V235I TM1	-0.9	3.12671	0.40707	1	0.05	0	-12.60591	10.80591
V235I TM2	44.04444	3.05917	20.36114	3.12901E-8	0.05	1	32.59139	55.4975
V235I TM3	8.17143	3.21146	3.59841	0.67901	0.05	0	-3.85177	20.19463
V235I TM4	1.17143	3.21146	0.51586	1	0.05	0	-10.85177	13.19463
V235I TM5	0.74286	3.21146	0.32713	1	0.05	0	-11.28034	12.76606
V235I TM6	0.725	3.12671	0.32792	1	0.05	0	-10.98091	12.43091
V235I TM7	49.4	3.00405	23.25601	0	0.05	1	38.15333	60.64667
V235I A66V	-2.11429	3.21146	0.93106	1	0.05	0	-14.13748	9.90891
V235I F69L	12.74286	3.21146	5.6115	0.02413	0.05	1	0.71966	24.76606
V235I A71G	2.02857	3.21146	0.89331	1	0.05	0	-9.99463	14.05177
V235I L72I	1.76667	3.3211	0.75229	1	0.05	0	-10.667	14.20033
V235I S73I	46.31429	3.21146	20.39516	3.14599E-8	0.05	1	34.29109	58.33748
V235I V76L	1.76667	3.3211	0.75229	1	0.05	0	-10.667	14.20033
V235I S77C	3.6	3.21146	1.58531	0.99999	0.05	0	-8.4232	15.6232
V235I I80M	-2.4	3.3211	1.02198	1	0.05	0	-14.83366	10.03366
V235I D217S	-1.11429	3.21146	0.49069	1	0.05	0	-13.13748	10.90891
V235I T222Y	63.45714	3.21146	27.94427	0	0.05	1	51.43394	75.48034
V235I F231T	-7.11429	3.21146	3.13288	0.88247	0.05	0	-19.13748	4.90891
V235I T234S	-0.4	3.3211	0.17033	1	0.05	0	-12.83366	12.03366
L238I HcCC	-4.86667	3.05917	2.24979	0.99761	0.05	0	-16.31972	6.58638
L238I N	-9.075	3.12671	4.10463	0.40558	0.05	0	-20.78091	2.63091
L238I TM1	-6.7	3.12671	3.03041	0.91273	0.05	0	-18.40591	5.00591
L238I TM2	38.24444	3.05917	17.67988	2.84591E-8	0.05	1	26.79139	49.6975
L238I TM3	2.37143	3.21146	1.04429	1	0.05	0	-9.65177	14.39463
L238I TM4	-4.62857	3.21146	2.03826	0.99948	0.05	0	-16.65177	7.39463
L238I TM5	-5.05714	3.21146	2.22699	0.99794	0.05	0	-17.08034	6.96606
L238I TM6	-5.075	3.12671	2.29543	0.9968	0.05	0	-16.78091	6.63091
L238I TM7	43.6	3.00405	20.52554	3.21043E-8	0.05	1	32.35333	54.84667
L238I A66V	-7.91429	3.21146	3.48517	0.73678	0.05	0	-19.93748	4.10891
L238I F69L	6.94286	3.21146	3.05739	0.90532	0.05	0	-5.08034	18.96606
L238I A71G	-3.77143	3.21146	1.6608	0.99999	0.05	0	-15.79463	8.25177
L238I L72I	-4.03333	3.3211	1.7175	0.99997	0.05	0	-16.467	8.40033
L238I S73I	40.51429	3.21146	17.84105	2.96016E-8	0.05	1	28.49109	52.53748
L238I V76L	-4.03333	3.3211	1.7175	0.99997	0.05	0	-16.467	8.40033
L238I S77C	-2.2	3.21146	0.9688	1	0.05	0	-14.2232	9.8232
L238I I80M	-8.2	3.3211	3.49178	0.73352	0.05	0	-20.63366	4.23366
L238I D217S	-6.91429	3.21146	3.04481	0.90882	0.05	0	-18.93748	5.10891
L238I T222Y	57.65714	3.21146	25.39015	0	0.05	1	45.63394	69.68034
L238I F231T	-12.91429	3.21146	5.68699	0.02011	0.05	1	-24.93748	-0.89109
L238I T234S	-6.2	3.3211	2.64013	0.9798	0.05	0	-18.63366	6.23366
L238I V235I	-5.8	3.46878	2.36465	0.99513	0.05	0	-18.78654	7.18654
I239L HcCC	5.08333	2.66504	2.69749	0.97401	0.05	0	-4.89416	15.06083
I239L N	0.875	2.74231	0.45124	1	0.05	0	-9.39176	11.14176
I239L TM1	3.25	2.74231	1.67603	0.99998	0.05	0	-7.01676	13.51676
I239L TM2	48.19444	2.66504	25.57452	0	0.05	1	38.21695	58.17194
I239L TM3	12.32143	2.83856	6.13873	0.00633	0.05	1	1.69432	22.94854
I239L TM4	5.32143	2.83856	2.65122	0.97877	0.05	0	-5.30568	15.94854
I239L TM5	4.89286	2.83856	2.4377	0.99263	0.05	0	-5.73425	15.51996
I239L TM6	4.875	2.74231	2.51405	0.98897	0.05	0	-5.39176	15.14176
I239L TM7	53.55	2.60158	29.10965	0	0.05	1	43.8101	63.2899
I239L A66V	2.03571	2.83856	1.01422	1	0.05	0	-8.59139	12.66282
I239L F69L	16.89286	2.83856	8.41628	5.07861E-6	0.05	1	6.26575	27.51996
I239L A71G	6.17857	2.83856	3.07826	0.89932	0.05	0	-4.44854	16.80568
I239L L72I	5.91667	2.96203	2.8249	0.95666	0.05	0	-5.1727	17.00604
I239L S73I	50.46429	2.83856	25.14209	0	0.05	1	39.83718	61.09139
I239L V76L	5.91667	2.96203	2.8249	0.95666	0.05	0	-5.1727	17.00604
I239L S77C	7.75	2.83856	3.86117	0.53587	0.05	0	-2.87711	18.37711
I239L I80M	1.75	2.96203	0.83553	1	0.05	0	-9.33937	12.83937
I239L D217S	3.03571	2.83856	1.51244	1	0.05	0	-7.59139	13.66282
I239L T222Y	67.60714	2.83856	33.68292	0	0.05	1	56.98004	78.23425
I239L F231T	-2.96429	2.83856	1.47685	1	0.05	0	-13.59139	7.66282
I239L T234S	3.75	2.96203	1.79043	0.99994	0.05	0	-7.33937	14.83937
I239L V235I	4.15	3.12671	1.87705	0.99987	0.05	0	-7.55591	15.85591
I239L L238I	9.95	3.12671	4.50039	0.22953	0.05	0	-1.75591	21.65591
S73I_T222Y	-70.66667	2.66504	37.49948	0	0.05	1	-80.64416	-60.68917
S73I_T222Y	-74.875	2.74231	38.61319	0	0.05	1	-85.14176	-64.60824
S73I_T222Y	-72.5	2.74231	37.38839	0	0.05	1	-82.76676	-62.23324
S73I_T222Y	-27.55556	2.66504	14.62244	0	0.05	1	-37.53305	-17.57806
S73I_T222Y	-63.42857	2.83856	31.60109	0	0.05	1	-74.05568	-52.80146
S73I_T222Y	-70.42857	2.83856	35.0886	0	0.05	1	-81.05568	-59.80146
S73I_T222Y	-70.85714	2.83856	35.30212	0	0.05	1	-81.48425	-60.23004
S73I_T222Y	-70.875	2.74231	36.55038	0	0.05	1	-81.14176	-60.60824
S73I_T222Y	-22.2	2.60158	12.06787	5.79467E-8	0.05	1	-31.9399	-12.4601
S73I_T222Y	-73.71429	2.83856	36.7256	0	0.05	1	-84.34139	-63.08718
S73I_T222Y	-58.85714	2.83856	29.32354	0	0.05	1	-69.48425	-48.23004
S73I_T222Y	-69.57143	2.83856	34.66156	0	0.05	1	-80.19854	-58.94432
S73I_T222Y	-69.83333	2.96203	33.34173	0	0.05	1	-80.9227	-58.74396
S73I_T222Y	-25.28571	2.83856	12.59773	0	0.05	1	-35.91282	-14.65861
S73I_T222Y	-69.83333	2.96203	33.34173	0	0.05	1	-80.9227	-58.74396
S73I_T222Y	-68	2.83856	33.87865	0	0.05	1	-78.62711	-57.37289
S73I_T222Y	-74	2.96203	35.3311	0	0.05	1	-85.08937	-62.91063
S73I_T222Y	-72.71429	2.83856	36.22738	0	0.05	1	-83.34139	-62.08718
S73I_T222Y	-8.14286	2.83856	4.0569	0.43025	0.05	0	-18.76996	2.48425
S73I_T222Y	-78.71429	2.83856	39.21667	0	0.05	1	-89.34139	-68.08718
S73I_T222Y	-72	2.96203	34.3762	0	0.05	1	-83.08937	-60.91063
S73I_T222Y	-71.6	3.12671	32.38472	0	0.05	1	-83.30591	-59.89409
S73I_T222Y	-65.8	3.12671	29.76138	0	0.05	1	-77.50591	-54.09409
S73I_T222Y	-75.75	2.74231	39.06442	0	0.05	1	-86.01676	-65.48324
F69L_S73I_T	-97.54167	2.66504	51.76077	0	0.05	1	-107.5191	-87.56417
F69L_S73I_T	-101.75	2.74231	52.47268	0	0.05	1	-112.0167	-91.48324
F69L_S73I_T	-99.375	2.74231	51.24788	0	0.05	1	-109.6417	-89.10824
F69L_S73I_T	-54.43056	2.66504	28.88374	0	0.05	1	-64.40805	-44.45306
F69L_S73I_T	-90.30357	2.83856	44.99063	0	0.05	1	-100.9306	-79.67646
F69L_S73I_T	-97.30357	2.83856	48.47814	0	0.05	1	-107.9306	-86.67646
F69L_S73I_T	-97.73214	2.83856	48.69166	0	0.05	1	-108.3592	-87.10504
F69L_S73I_T	-97.75	2.74231	50.40987	0	0.05	1	-108.0167	-87.48324
F69L_S73I_T	-49.075	2.60158	26.67705	0	0.05	1	-58.8149	-39.3351
F69L_S73I_T	-100.5892	2.83856	50.11514	0	0.05	1	-111.2163	-89.96218
F69L_S73I_T	-85.73214	2.83856	42.71308	0	0.05	1	-96.35925	-75.10504
F69L_S73I_T	-96.44643	2.83856	48.0511	0	0.05	1	-107.0735	-85.81932
F69L_S73I_T	-96.70833	2.96203	46.17313	0	0.05	1	-107.7977	-85.61896
F69L_S73I_T	-52.16071	2.83856	25.98727	0	0.05	1	-62.78782	-41.53361
F69L_S73I_T	-96.70833	2.96203	46.17313	0	0.05	1	-107.7977	-85.61896
F69L_S73I_T	-94.875	2.83856	47.26819	0	0.05	1	-105.5021	-84.24789
F69L_S73I_T	-100.875	2.96203	48.16249	0	0.05	1	-111.9643	-89.78563
F69L_S73I_T	-99.58929	2.83856	49.61692	0	0.05	1	-110.2163	-88.96218
F69L_S73I_T	-35.01786	2.83856	17.44644	0	0.05	1	-45.64496	-24.39075
F69L_S73I_T	-105.5892	2.83856	52.60621	0	0.05	1	-116.2163	-94.96218
F69L_S73I_T	-98.875	2.96203	47.2076	0	0.05	1	-109.9643	-87.78563
F69L_S73I_T	-98.475	3.12671	44.5403	0	0.05	1	-110.1809	-86.76909
F69L_S73I_T	-92.675	3.12671	41.91695	0	0.05	1	-104.3809	-80.96909
F69L_S73I_T	-102.625	2.74231	52.92392	0	0.05	1	-112.8917	-92.35824
F69L_S73I_T	-26.875	2.74231	13.85949	0	0.05	1	-37.14176	-16.60824

Sig equals 1 indicates that the difference of the means is significant at the 0.05 level.
Sig equals 0 indicates that the difference of the means is not significant at the 0.05 level.

ANOVAOneWay (8/4/2022 14:30:47)

Descriptive Statistics

	N Analysis	N Missing	Mean	Standard Deviation	SE of Mean
HcCCR	9	0	45.26667	4.06202	1.35401
N	8	0	49.475	4.99821	1.76714
TM1	8	0	47.1	6.39196	2.2599
TM2	9	0	2.15556	5.19882	1.73294
TM3	7	0	38.02857	4.64963	1.75739
TM4	7	0	45.02857	5.287	1.9983
TM5	7	0	45.45714	5.63999	2.13172
TM6	8	0	45.475	5.40998	1.91272
TM7	10	0	-3.2	11.01312	3.48266
A66V	7	0	48.31429	2.75162	1.04002
F69L	7	0	33.45714	5.08031	1.92018
A71G	7	0	44.17143	4.35343	1.64544
L72I	6	0	44.43333	2.40139	0.98036
S73I	7	0	-0.11429	3.77334	1.42619
V76L	6	0	44.43333	4.40076	1.7966
S77C	7	0	42.6	4.24264	1.60357
I80M	6	0	48.6	5.86515	2.39444
D217S	7	0	47.31429	3.03942	1.14879
T222Y	7	0	-17.25714	3.67099	1.3875
F231T	7	0	53.31429	5.2825	1.9966
T234S	6	0	46.6	6.57267	2.68328
V235I	5	0	46.2	8.59069	3.84187
L238I	5	0	40.4	6.30079	2.8178
I239L	8	0	50.35	3.41216	1.20638
S73I_T222Y	8	0	-25.4	5.15475	1.82248
F69L_S73I_T222	8	0	-52.275	5.38351	1.90336

One Way ANOVA

Overall ANOVA

	DF	Sum of Squares	Mean Square	F Value	Prob>F
Model	25	153975.12866	6159.00515	204.74729	9.48544E-109
Error	161	4843.04246	30.08101		
Total	186	158818.17112			

Null Hypothesis: The means of all levels are equal.
Alternative Hypothesis: The means of one or more levels are different.
At the 0.01 level, the population means are significantly different.

Fit Statistics

	R-Square	Coeff Var	Root MSE	Data Mean
	0.96951	0.18606	5.48462	29.47701

Means Comparisons

Tukey Test

	MeanDiff	SEM	q Value	Prob	Alpha	Sig	LCL	UCL
N HcCCR	4.20833	2.66504	2.23316	0.99786	0.01	0	-7.03265	15.44932
TM1 HcCCR	1.83333	2.66504	0.97286	1	0.01	0	-9.40765	13.07432
TM1 N	-2.375	2.74231	1.22479	1	0.01	0	-13.94188	9.19188
TM2 HcCCR	-43.11111	2.58547	23.58111	0	0.01	1	-54.01647	-32.20575
TM2 N	-47.31944	2.66504	25.1102	0	0.01	1	-58.56043	-36.07846
TM2 TM1	-44.94444	2.66504	23.8499	0	0.01	1	-56.18543	-33.70346
TM3 HcCCR	-7.2381	2.76399	3.70342	0.62266	0.01	0	-18.89641	4.42022
TM3 N	-11.44643	2.83856	5.70279	0.01935	0.01	0	-23.41929	0.52643
TM3 TM1	-9.07143	2.83856	4.51953	0.22252	0.01	0	-21.04429	2.90143
TM3 TM2	35.87302	2.76399	18.35469	4.72423E-7	0.01	1	24.2147	47.53133
TM4 HcCCR	-0.2381	2.76399	0.12182	1	0.01	0	-11.89641	11.42022
TM4 N	-4.44643	2.83856	2.21528	0.9981	0.01	0	-16.41929	7.52643
TM4 TM1	-2.07143	2.83856	1.03202	1	0.01	0	-14.04429	9.90143
TM4 TM2	42.87302	2.76399	21.93629	0	0.01	1	31.2147	54.53133
TM4 TM3	7	2.93165	3.37677	0.78784	0.01	0	-5.36551	19.36551
TM5 HcCCR	0.19048	2.76399	0.09746	1	0.01	0	-11.46784	11.84879
TM5 N	-4.01786	2.83856	2.00176	0.99961	0.01	0	-15.99071	7.955
TM5 TM1	-1.64286	2.83856	0.8185	1	0.01	0	-13.61571	10.33
TM5 TM2	43.30159	2.76399	22.15557	0	0.01	1	31.64327	54.95991
TM5 TM3	7.42857	2.93165	3.58351	0.68682	0.01	0	-4.93694	19.79409
TM5 TM4	0.42857	2.93165	0.20674	1	0.01	0	-11.93694	12.79409
TM6 HcCCR	0.20833	2.66504	0.11055	1	0.01	0	-11.03265	11.44932
TM6 N	-4	2.74231	2.06281	0.99937	0.01	0	-15.56688	7.56688
TM6 TM1	-1.625	2.74231	0.83802	1	0.01	0	-13.19188	9.94188
TM6 TM2	43.31944	2.66504	22.98759	0	0.01	1	32.07846	54.56043
TM6 TM3	7.44643	2.83856	3.70993	0.61911	0.01	0	-4.52643	19.41929
TM6 TM4	0.44643	2.83856	0.22242	1	0.01	0	-11.52643	12.41929
TM6 TM5	0.01786	2.83856	0.0089	1	0.01	0	-11.955	11.99071
TM7 HcCCR	-48.46667	2.52001	27.19922	0	0.01	1	-59.09589	-37.83744
TM7 N	-52.675	2.60158	28.634	0	0.01	1	-63.6483	-41.7017
TM7 TM1	-50.3	2.60158	27.34296	0	0.01	1	-61.2733	-39.3267
TM7 TM2	-5.35556	2.52001	3.00551	0.91921	0.01	0	-15.98478	5.27367
TM7 TM3	-41.22857	2.70285	21.57206	0	0.01	1	-52.62901	-29.82813
TM7 TM4	-48.22857	2.70285	25.23468	0	0.01	1	-59.62901	-36.82813
TM7 TM5	-48.65714	2.70285	25.45892	0	0.01	1	-60.05758	-37.2567
TM7 TM6	-48.675	2.60158	26.45961	0	0.01	1	-59.6483	-37.7017
A66V HcCC	3.04762	2.76399	1.55934	1	0.01	0	-8.6107	14.70594
A66V N	-1.16071	2.83856	0.57829	1	0.01	0	-13.13357	10.81214
A66V TM1	1.21429	2.83856	0.60498	1	0.01	0	-10.75857	13.18714
A66V TM2	46.15873	2.76399	23.61745	0	0.01	1	34.50041	57.81705
A66V TM3	10.28571	2.93165	4.96178	0.10019	0.01	0	-2.0798	22.65123
A66V TM4	3.28571	2.93165	1.58501	0.99999	0.01	0	-9.0798	15.65123
A66V TM5	2.85714	2.93165	1.37827	1	0.01	0	-9.50837	15.22266
A66V TM6	2.83929	2.83856	1.41458	1	0.01	0	-9.13357	14.81214
A66V TM7	51.51429	2.70285	26.95386	0	0.01	1	40.11385	62.91473
F69L HcCCR	-11.80952	2.76399	6.04243	0.00817	0.01	1	-23.46784	-0.15121
F69L N	-16.01786	2.83856	7.98034	2.2742E-5	0.01	1	-27.99071	-4.045
F69L TM1	-13.64286	2.83856	6.79708	9.85542E-4	0.01	1	-25.61571	-1.67
F69L TM2	31.30159	2.76399	16.01568	0	0.01	1	19.64327	42.95991
F69L TM3	-4.57143	2.93165	2.20523	0.99822	0.01	0	-16.93694	7.79409
F69L TM4	-11.57143	2.93165	5.582	0.02589	0.01	0	-23.93694	0.79409
F69L TM5	-12	2.93165	5.78874	0.01565	0.01	0	-24.36551	0.36551
F69L TM6	-12.01786	2.83856	5.98748	0.00944	0.01	1	-23.99071	-0.045
F69L TM7	36.65714	2.70285	19.18015	1.5008E-7	0.01	1	25.2567	48.05758
F69L A66V	-14.85714	2.93165	7.16701	3.20034E-4	0.01	1	-27.22266	-2.49163
A71G HcCC	-1.09524	2.76399	0.56039	1	0.01	0	-12.75356	10.56308
A71G N	-5.30357	2.83856	2.64232	0.9796	0.01	0	-17.27643	6.66929
A71G TM1	-2.92857	2.83856	1.45906	1	0.01	0	-14.90143	9.04429
A71G TM2	42.01587	2.76399	21.49772	0	0.01	1	30.35755	53.67419
A71G TM3	6.14286	2.93165	2.96328	0.92946	0.01	0	-6.22266	18.50837
A71G TM4	-0.85714	2.93165	0.41348	1	0.01	0	-13.22266	11.50837
A71G TM5	-1.28571	2.93165	0.62022	1	0.01	0	-13.65123	11.0798
A71G TM6	-1.30357	2.83856	0.64946	1	0.01	0	-13.27643	10.66929
A71G TM7	47.37143	2.70285	24.78619	0	0.01	1	35.97099	58.77187
A71G A66V	-4.14286	2.93165	1.99849	0.99962	0.01	0	-16.50837	8.22266
A71G F69L	10.71429	2.93165	5.16852	0.06564	0.01	0	-1.65123	23.0798
L72I HcCCR	-0.83333	2.89065	0.4077	1	0.01	0	-13.02589	11.35923
L72I N	-5.04167	2.96203	2.40713	0.99378	0.01	0	-17.53532	7.45199
L72I TM1	-2.66667	2.96203	1.27319	1	0.01	0	-15.16032	9.82699
L72I TM2	42.27778	2.89065	20.68389	0	0.01	1	30.08522	54.47034
L72I TM3	6.40476	3.05136	2.96842	0.92826	0.01	0	-6.46567	19.2752
L72I TM4	-0.59524	3.05136	0.27588	1	0.01	0	-13.46567	12.2752
L72I TM5	-1.02381	3.05136	0.47451	1	0.01	0	-13.89424	11.84663
L72I TM6	-1.04167	2.96203	0.49734	1	0.01	0	-13.53532	11.45199
L72I TM7	47.63333	2.83224	23.78458	0	0.01	1	35.68711	59.57955
L72I A66V	-3.88095	3.05136	1.79871	0.99994	0.01	0	-16.75139	8.98948
L72I F69L	10.97619	3.05136	5.08714	0.0778	0.01	0	-1.89424	23.84663
L72I A71G	0.2619	3.05136	0.12139	1	0.01	0	-12.60853	13.13234
S73I HcCCR	-45.38095	2.76399	23.21949	0	0.01	1	-57.03927	-33.72263
S73I N	-49.58929	2.83856	24.70615	0	0.01	1	-61.56214	-37.61643
S73I TM1	-47.21429	2.83856	23.52289	0	0.01	1	-59.18714	-35.24143
S73I TM2	-2.26984	2.76399	1.16138	1	0.01	0	-13.92816	9.38848
S73I TM3	-38.14286	2.93165	18.39992	4.75842E-7	0.01	1	-50.50837	-25.77734
S73I TM4	-45.14286	2.93165	21.77669	0	0.01	1	-57.50837	-32.77734
S73I TM5	-45.57143	2.93165	21.98343	0	0.01	1	-57.93694	-33.20591
S73I TM6	-45.58929	2.83856	22.71329	0	0.01	1	-57.56214	-33.61643
S73I TM7	3.08571	2.70285	1.61454	0.99999	0.01	0	-8.31473	14.48615
S73I A66V	-48.42857	2.93165	23.3617	0	0.01	1	-60.79409	-36.06306
S73I F69L	-33.57143	2.93165	16.19469	0	0.01	1	-45.93694	-21.20591
S73I A71G	-44.28571	2.93165	21.36321	0	0.01	1	-56.65123	-31.9202
S73I L72I	-44.54762	3.05136	20.64649	0	0.01	1	-57.41805	-31.67718
V76L HcCCR	-0.83333	2.89065	0.4077	1	0.01	0	-13.02589	11.35923
V76L N	-5.04167	2.96203	2.40713	0.99378	0.01	0	-17.53532	7.45199
V76L TM1	-2.66667	2.96203	1.27319	1	0.01	0	-15.16032	9.82699
V76L TM2	42.27778	2.89065	20.68389	0	0.01	1	30.08522	54.47034
V76L TM3	6.40476	3.05136	2.96842	0.92826	0.01	0	-6.46567	19.2752
V76L TM4	-0.59524	3.05136	0.27588	1	0.01	0	-13.46567	12.2752
V76L TM5	-1.02381	3.05136	0.47451	1	0.01	0	-13.89424	11.84663
V76L TM6	-1.04167	2.96203	0.49734	1	0.01	0	-13.53532	11.45199
V76L TM7	47.63333	2.83224	23.78458	0	0.01	1	35.68711	59.57955
V76L A66V	-3.88095	3.05136	1.79871	0.99994	0.01	0	-16.75139	8.98948
V76L F69L	10.97619	3.05136	5.08714	0.0778	0.01	0	-1.89424	23.84663
V76L A71G	0.2619	3.05136	0.12139	1	0.01	0	-12.60853	13.13234
V76L L72I	-7.10543E	3.16654	3.17336E	1	0.01	0	-13.35628	13.35628
V76L S73I	44.54762	3.05136	20.64649	0	0.01	1	31.67718	57.41805
S77C HcCC	-2.66667	2.76399	1.36442	1	0.01	0	-14.32498	8.99165
S77C N	-6.875	2.83856	3.42523	0.7656	0.01	0	-18.84786	5.09786
S77C TM1	-4.5	2.83856	2.24197	0.99773	0.01	0	-16.47286	7.47286
S77C TM2	40.44444	2.76399	20.69369	0	0.01	1	28.78613	52.10276
S77C TM3	4.57143	2.93165	2.20523	0.99822	0.01	0	-7.79409	16.93694
S77C TM4	-2.42857	2.93165	1.17153	1	0.01	0	-14.79409	9.93694
S77C TM5	-2.85714	2.93165	1.37827	1	0.01	0	-15.22266	9.50837
S77C TM6	-2.875	2.83856	1.43237	1	0.01	0	-14.84786	9.09786
S77C TM7	45.8	2.70285	23.96397	0	0.01	1	34.39956	57.20044
S77C A66V	-5.71429	2.93165	2.75654	0.9668	0.01	0	-18.0798	6.65123
S77C F69L	9.14286	2.93165	4.41047	0.26441	0.01	0	-3.22266	21.50837
S77C A71G	-1.57143	2.93165	0.75805	1	0.01	0	-13.93694	10.79409
S77C L72I	-1.83333	3.05136	0.8497	1	0.01	0	-14.70377	11.0371
S77C S73I	42.71429	2.93165	20.60516	0	0.01	1	30.34877	55.0798
S77C V76L	-1.83333	3.05136	0.8497	1	0.01	0	-14.70377	11.0371
I80M HcCCR	3.33333	2.89065	1.63079	0.99999	0.01	0	-8.85923	15.52589
I80M N	-0.875	2.96203	0.41777	1	0.01	0	-13.36866	11.61866
I80M TM1	1.5	2.96203	0.71617	1	0.01	0	-10.99366	13.99366
I80M TM2	46.44444	2.89065	22.72238	0	0.01	1	34.25188	58.63701
I80M TM3	10.57143	3.05136	4.89954	0.11311	0.01	0	-2.29901	23.44186
I80M TM4	3.57143	3.05136	1.65525	0.99999	0.01	0	-9.29901	16.44186
I80M TM5	3.14286	3.05136	1.45662	1	0.01	0	-9.72758	16.01329
I80M TM6	3.125	2.96203	1.49202	1	0.01	0	-9.36866	15.61866
I80M TM7	51.8	2.83224	25.8651	0	0.01	1	39.85378	63.74622
I80M A66V	0.28571	3.05136	0.13242	1	0.01	0	-12.58472	13.15615
I80M F69L	15.14286	3.05136	7.01826	5.06251E-4	0.01	1	2.27242	28.01329
I80M A71G	4.42857	3.05136	2.05251	0.99942	0.01	0	-8.44186	17.29901
I80M L72I	4.16667	3.16654	1.86088	0.99989	0.01	0	-9.18961	17.52295
I80M S73I	48.71429	3.05136	22.57762	0	0.01	1	35.84385	61.58472
I80M V76L	4.16667	3.16654	1.86088	0.99989	0.01	0	-9.18961	17.52295
I80M S77C	6	3.05136	2.78082	0.96342	0.01	0	-6.87043	18.87043
D217S HcCC	2.04762	2.76399	1.04768	1	0.01	0	-9.6107	13.70594
D217S N	-2.16071	2.83856	1.0765	1	0.01	0	-14.13357	9.81214
D217S TM1	0.21429	2.83856	0.10676	1	0.01	0	-11.75857	12.18714
D217S TM2	45.15873	2.76399	23.10579	0	0.01	1	33.50041	56.81705
D217S TM3	9.28571	2.93165	4.47938	0.23739	0.01	0	-3.0798	21.65123
D217S TM4	2.28571	2.93165	1.10262	1	0.01	0	-10.0798	14.65123
D217S TM5	1.85714	2.93165	0.89588	1	0.01	0	-10.50837	14.22266
D217S TM6	1.83929	2.83856	0.91636	1	0.01	0	-10.13357	13.81214
D217S TM7	50.51429	2.70285	26.43063	0	0.01	1	39.11385	61.91473
D217S A66V	-1	2.93165	0.4824	1	0.01	0	-13.36551	11.36551
D217S F69L	13.85714	2.93165	6.68462	0.00137	0.01	1	1.49163	26.22266
D217S A71G	3.14286	2.93165	1.5161	1	0.01	0	-9.22266	15.50837
D217S L72I	2.88095	3.05136	1.33524	1	0.01	0	-9.98948	15.75139
D217S S73I	47.42857	2.93165	22.87931	0	0.01	1	35.06306	59.79409
D217S V76L	2.88095	3.05136	1.33524	1	0.01	0	-9.98948	15.75139
D217S S77C	4.71429	2.93165	2.27415	0.9972	0.01	0	-7.65123	17.0798
D217S I80M	-1.28571	3.05136	0.59589	1	0.01	0	-14.15615	11.58472
T222Y HcCC	-62.52381	2.76399	31.99076	0	0.01	1	-74.18213	-50.86549
T222Y N	-66.73214	2.83856	33.24698	0	0.01	1	-78.705	-54.75929
T222Y TM1	-64.35714	2.83856	32.06372	0	0.01	1	-76.33	-52.38429
T222Y TM2	-19.4127	2.76399	9.93265	4.21357E-8	0.01	1	-31.07102	-7.75438
T222Y TM3	-55.28571	2.93165	26.66955	0	0.01	1	-67.65123	-42.9202
T222Y TM4	-62.28571	2.93165	30.04632	0	0.01	1	-74.65123	-49.9202
T222Y TM5	-62.71429	2.93165	30.25306	0	0.01	1	-75.0798	-50.34877
T222Y TM6	-62.73214	2.83856	31.25412	0	0.01	1	-74.705	-50.75929
T222Y TM7	-14.05714	2.70285	7.35513	1.7711E-4	0.01	1	-25.45758	-2.6567
T222Y A66V	-65.57143	2.93165	31.63133	0	0.01	1	-77.93694	-53.20591
T222Y F69L	-50.71429	2.93165	24.46432	0	0.01	1	-63.0798	-38.34877
T222Y A71G	-61.42857	2.93165	29.63284	0	0.01	1	-73.79409	-49.06306
T222Y L72I	-61.69048	3.05136	28.59169	0	0.01	1	-74.56091	-48.82004
T222Y S73I	-17.14286	2.93165	8.26963	8.44153E-6	0.01	1	-29.50837	-4.77734
T222Y V76L	-61.69048	3.05136	28.59169	0	0.01	1	-74.56091	-48.82004
T222Y S77C	-59.85714	2.93165	28.87479	0	0.01	1	-72.22266	-47.49163
T222Y I80M	-65.85714	3.05136	30.52282	0	0.01	1	-78.72758	-52.98671
T222Y D217	-64.57143	2.93165	31.14894	0	0.01	1	-76.93694	-52.20591
F231T HcCC	8.04762	2.76399	4.11762	0.39897	0.01	0	-3.6107	19.70594
F231T N	3.83929	2.83856	1.91279	0.99982	0.01	0	-8.13357	15.81214
F231T TM1	6.21429	2.83856	3.09605	0.89401	0.01	0	-5.75857	18.18714
F231T TM2	51.15873	2.76399	26.17573	0	0.01	1	39.50041	62.81705
F231T TM3	15.28571	2.93165	7.37375	1.66921E-4	0.01	1	2.9202	27.65123
F231T TM4	8.28571	2.93165	3.99699	0.46194	0.01	0	-4.0798	20.65123
F231T TM5	7.85714	2.93165	3.79025	0.57499	0.01	0	-4.50837	20.22266
F231T TM6	7.83929	2.83856	3.90565	0.51143	0.01	0	-4.13357	19.81214
F231T TM7	56.51429	2.70285	29.57002	0	0.01	1	45.11385	67.91473
F231T A66V	5	2.93165	2.41198	0.99361	0.01	0	-7.36551	17.36551
F231T F69L	19.85714	2.93165	9.57899	9.59938E-8	0.01	1	7.49163	32.22266
F231T A71G	9.14286	2.93165	4.41047	0.26441	0.01	0	-3.22266	21.50837
F231T L72I	8.88095	3.05136	4.11606	0.39976	0.01	0	-3.98948	21.75139
F231T S73I	53.42857	2.93165	25.77368	0	0.01	1	41.06306	65.79409
F231T V76L	8.88095	3.05136	4.11606	0.39976	0.01	0	-3.98948	21.75139
F231T S77C	10.71429	2.93165	5.16852	0.06564	0.01	0	-1.65123	23.0798
F231T I80M	4.71429	3.05136	2.18493	0.99845	0.01	0	-8.15615	17.58472
F231T D217	6	2.93165	2.89437	0.94418	0.01	0	-6.36551	18.36551
F231T T222	70.57143	2.93165	34.04331	0	0.01	1	58.20591	82.93694
T234S HcCC	1.33333	2.89065	0.65232	1	0.01	0	-10.85923	13.52589
T234S N	-2.875	2.96203	1.37266	1	0.01	0	-15.36866	9.61866
T234S TM1	-0.5	2.96203	0.23872	1	0.01	0	-12.99366	11.99366
T234S TM2	44.44444	2.89065	21.7439	0	0.01	1	32.25188	56.63701
T234S TM3	8.57143	3.05136	3.9726	0.47504	0.01	0	-4.29901	21.44186
T234S TM4	1.57143	3.05136	0.72831	1	0.01	0	-11.29901	14.44186
T234S TM5	1.14286	3.05136	0.52968	1	0.01	0	-11.72758	14.01329
T234S TM6	1.125	2.96203	0.53713	1	0.01	0	-11.36866	13.61866
T234S TM7	49.8	2.83224	24.86645	0	0.01	1	37.85378	61.74622
T234S A66V	-1.71429	3.05136	0.79452	1	0.01	0	-14.58472	11.15615
T234S F69L	13.14286	3.05136	6.09132	0.00718	0.01	1	0.27242	26.01329
T234S A71G	2.42857	3.05136	1.12557	1	0.01	0	-10.44186	15.29901
T234S L72I	2.16667	3.16654	0.96766	1	0.01	0	-11.18961	15.52295
T234S S73I	46.71429	3.05136	21.65068	0	0.01	1	33.84385	59.58472
T234S V76L	2.16667	3.16654	0.96766	1	0.01	0	-11.18961	15.52295
T234S S77C	4	3.05136	1.85388	0.99989	0.01	0	-8.87043	16.87043
T234S I80M	-2	3.16654	0.89322	1	0.01	0	-15.35628	11.35628
T234S D217	-0.71429	3.05136	0.33105	1	0.01	0	-13.58472	12.15615
T234S T222	63.85714	3.05136	29.59588	0	0.01	1	50.98671	76.72758
T234S F231	-6.71429	3.05136	3.11187	0.88914	0.01	0	-19.58472	6.15615
V235I HcCC	0.93333	3.05917	0.43147	1	0.01	0	-11.97006	13.83673
V235I N	-3.275	3.12671	1.48128	1	0.01	0	-16.46327	9.91327
V235I TM1	-0.9	3.12671	0.40707	1	0.01	0	-14.08827	12.28827
V235I TM2	44.04444	3.05917	20.36114	3.12901E-8	0.01	1	31.14105	56.94784
V235I TM3	8.17143	3.21146	3.59841	0.67901	0.01	0	-5.37431	21.71717
V235I TM4	1.17143	3.21146	0.51586	1	0.01	0	-12.37431	14.71717
V235I TM5	0.74286	3.21146	0.32713	1	0.01	0	-12.80288	14.2886
V235I TM6	0.725	3.12671	0.32792	1	0.01	0	-12.46327	13.91327
V235I TM7	49.4	3.00405	23.25601	0	0.01	1	36.72912	62.07088
V235I A66V	-2.11429	3.21146	0.93106	1	0.01	0	-15.66003	11.43146
V235I F69L	12.74286	3.21146	5.6115	0.02413	0.01	0	-0.80288	26.2886
V235I A71G	2.02857	3.21146	0.89331	1	0.01	0	-11.51717	15.57431
V235I L72I	1.76667	3.3211	0.75229	1	0.01	0	-12.24152	15.77485
V235I S73I	46.31429	3.21146	20.39516	3.14599E-8	0.01	1	32.76854	59.86003
V235I V76L	1.76667	3.3211	0.75229	1	0.01	0	-12.24152	15.77485
V235I S77C	3.6	3.21146	1.58531	0.99999	0.01	0	-9.94574	17.14574
V235I I80M	-2.4	3.3211	1.02198	1	0.01	0	-16.40819	11.60819
V235I D217S	-1.11429	3.21146	0.49069	1	0.01	0	-14.66003	12.43146
V235I T222Y	63.45714	3.21146	27.94427	0	0.01	1	49.9114	77.00288
V235I F231T	-7.11429	3.21146	3.13288	0.88247	0.01	0	-20.66003	6.43146
V235I T234S	-0.4	3.3211	0.17033	1	0.01	0	-14.40819	13.60819
L238I HcCC	-4.86667	3.05917	2.24979	0.99761	0.01	0	-17.77006	8.03673
L238I N	-9.075	3.12671	4.10463	0.40558	0.01	0	-22.26327	4.11327
L238I TM1	-6.7	3.12671	3.03041	0.91273	0.01	0	-19.88827	6.48827
L238I TM2	38.24444	3.05917	17.67988	2.84591E-8	0.01	1	25.34105	51.14784
L238I TM3	2.37143	3.21146	1.04429	1	0.01	0	-11.17431	15.91717
L238I TM4	-4.62857	3.21146	2.03826	0.99948	0.01	0	-18.17431	8.91717
L238I TM5	-5.05714	3.21146	2.22699	0.99794	0.01	0	-18.60288	8.4886
L238I TM6	-5.075	3.12671	2.29543	0.9968	0.01	0	-18.26327	8.11327
L238I TM7	43.6	3.00405	20.52554	3.21043E-8	0.01	1	30.92912	56.27088
L238I A66V	-7.91429	3.21146	3.48517	0.73678	0.01	0	-21.46003	5.63146
L238I F69L	6.94286	3.21146	3.05739	0.90532	0.01	0	-6.60288	20.4886
L238I A71G	-3.77143	3.21146	1.6608	0.99999	0.01	0	-17.31717	9.77431
L238I L72I	-4.03333	3.3211	1.7175	0.99997	0.01	0	-18.04152	9.97485
L238I S73I	40.51429	3.21146	17.84105	2.96016E-8	0.01	1	26.96854	54.06003
L238I V76L	-4.03333	3.3211	1.7175	0.99997	0.01	0	-18.04152	9.97485
L238I S77C	-2.2	3.21146	0.9688	1	0.01	0	-15.74574	11.34574
L238I I80M	-8.2	3.3211	3.49178	0.73352	0.01	0	-22.20819	5.80819
L238I D217S	-6.91429	3.21146	3.04481	0.90882	0.01	0	-20.46003	6.63146
L238I T222Y	57.65714	3.21146	25.39015	0	0.01	1	44.1114	71.20288
L238I F231T	-12.91429	3.21146	5.68699	0.02011	0.01	0	-26.46003	0.63146
L238I T234S	-6.2	3.3211	2.64013	0.9798	0.01	0	-20.20819	7.80819
L238I V235I	-5.8	3.46878	2.36465	0.99513	0.01	0	-20.43107	8.83107
I239L HcCC	5.08333	2.66504	2.69749	0.97401	0.01	0	-6.15765	16.32432
I239L N	0.875	2.74231	0.45124	1	0.01	0	-10.69188	12.44188
I239L TM1	3.25	2.74231	1.67603	0.99998	0.01	0	-8.31688	14.81688
I239L TM2	48.19444	2.66504	25.57452	0	0.01	1	36.95346	59.43543
I239L TM3	12.32143	2.83856	6.13873	0.00633	0.01	1	0.34857	24.29429
I239L TM4	5.32143	2.83856	2.65122	0.97877	0.01	0	-6.65143	17.29429
I239L TM5	4.89286	2.83856	2.4377	0.99263	0.01	0	-7.08	16.86571
I239L TM6	4.875	2.74231	2.51405	0.98897	0.01	0	-6.69188	16.44188
I239L TM7	53.55	2.60158	29.10965	0	0.01	1	42.5767	64.5233
I239L A66V	2.03571	2.83856	1.01422	1	0.01	0	-9.93714	14.00857
I239L F69L	16.89286	2.83856	8.41628	5.07861E-6	0.01	1	4.92	28.86571
I239L A71G	6.17857	2.83856	3.07826	0.89932	0.01	0	-5.79429	18.15143
I239L L72I	5.91667	2.96203	2.8249	0.95666	0.01	0	-6.57699	18.41032
I239L S73I	50.46429	2.83856	25.14209	0	0.01	1	38.49143	62.43714
I239L V76L	5.91667	2.96203	2.8249	0.95666	0.01	0	-6.57699	18.41032
I239L S77C	7.75	2.83856	3.86117	0.53587	0.01	0	-4.22286	19.72286
I239L I80M	1.75	2.96203	0.83553	1	0.01	0	-10.74366	14.24366
I239L D217S	3.03571	2.83856	1.51244	1	0.01	0	-8.93714	15.00857
I239L T222Y	67.60714	2.83856	33.68292	0	0.01	1	55.63429	79.58
I239L F231T	-2.96429	2.83856	1.47685	1	0.01	0	-14.93714	9.00857
I239L T234S	3.75	2.96203	1.79043	0.99994	0.01	0	-8.74366	16.24366
I239L V235I	4.15	3.12671	1.87705	0.99987	0.01	0	-9.03827	17.33827
I239L L238I	9.95	3.12671	4.50039	0.22953	0.01	0	-3.23827	23.13827
S73I_T222Y	-70.66667	2.66504	37.49948	0	0.01	1	-81.90765	-59.42568
S73I_T222Y	-74.875	2.74231	38.61319	0	0.01	1	-86.44188	-63.30812
S73I_T222Y	-72.5	2.74231	37.38839	0	0.01	1	-84.06688	-60.93312
S73I_T222Y	-27.55556	2.66504	14.62244	0	0.01	1	-38.79654	-16.31457
S73I_T222Y	-63.42857	2.83856	31.60109	0	0.01	1	-75.40143	-51.45571
S73I_T222Y	-70.42857	2.83856	35.0886	0	0.01	1	-82.40143	-58.45571
S73I_T222Y	-70.85714	2.83856	35.30212	0	0.01	1	-82.83	-58.88429
S73I_T222Y	-70.875	2.74231	36.55038	0	0.01	1	-82.44188	-59.30812
S73I_T222Y	-22.2	2.60158	12.06787	5.79467E-8	0.01	1	-33.1733	-11.2267
S73I_T222Y	-73.71429	2.83856	36.7256	0	0.01	1	-85.68714	-61.74143
S73I_T222Y	-58.85714	2.83856	29.32354	0	0.01	1	-70.83	-46.88429
S73I_T222Y	-69.57143	2.83856	34.66156	0	0.01	1	-81.54429	-57.59857
S73I_T222Y	-69.83333	2.96203	33.34173	0	0.01	1	-82.32699	-57.33968
S73I_T222Y	-25.28571	2.83856	12.59773	0	0.01	1	-37.25857	-13.31286
S73I_T222Y	-69.83333	2.96203	33.34173	0	0.01	1	-82.32699	-57.33968
S73I_T222Y	-68	2.83856	33.87865	0	0.01	1	-79.97286	-56.02714
S73I_T222Y	-74	2.96203	35.3311	0	0.01	1	-86.49366	-61.50634
S73I_T222Y	-72.71429	2.83856	36.22738	0	0.01	1	-84.68714	-60.74143
S73I_T222Y	-8.14286	2.83856	4.0569	0.43025	0.01	0	-20.11571	3.83
S73I_T222Y	-78.71429	2.83856	39.21667	0	0.01	1	-90.68714	-66.74143
S73I_T222Y	-72	2.96203	34.3762	0	0.01	1	-84.49366	-59.50634
S73I_T222Y	-71.6	3.12671	32.38472	0	0.01	1	-84.78827	-58.41173
S73I_T222Y	-65.8	3.12671	29.76138	0	0.01	1	-78.98827	-52.61173
S73I_T222Y	-75.75	2.74231	39.06442	0	0.01	1	-87.31688	-64.18312
F69L_S73I_T	-97.54167	2.66504	51.76077	0	0.01	1	-108.7826	-86.30068
F69L_S73I_T	-101.75	2.74231	52.47268	0	0.01	1	-113.3168	-90.18312
F69L_S73I_T	-99.375	2.74231	51.24788	0	0.01	1	-110.9418	-87.80812
F69L_S73I_T	-54.43056	2.66504	28.88374	0	0.01	1	-65.67154	-43.18957
F69L_S73I_T	-90.30357	2.83856	44.99063	0	0.01	1	-102.2764	-78.33071
F69L_S73I_T	-97.30357	2.83856	48.47814	0	0.01	1	-109.2764	-85.33071
F69L_S73I_T	-97.73214	2.83856	48.69166	0	0.01	1	-109.705	-85.75929
F69L_S73I_T	-97.75	2.74231	50.40987	0	0.01	1	-109.3168	-86.18312
F69L_S73I_T	-49.075	2.60158	26.67705	0	0.01	1	-60.0483	-38.1017
F69L_S73I_T	-100.5892	2.83856	50.11514	0	0.01	1	-112.5621	-88.61643
F69L_S73I_T	-85.73214	2.83856	42.71308	0	0.01	1	-97.705	-73.75929
F69L_S73I_T	-96.44643	2.83856	48.0511	0	0.01	1	-108.4192	-84.47357
F69L_S73I_T	-96.70833	2.96203	46.17313	0	0.01	1	-109.2019	-84.21468
F69L_S73I_T	-52.16071	2.83856	25.98727	0	0.01	1	-64.13357	-40.18786
F69L_S73I_T	-96.70833	2.96203	46.17313	0	0.01	1	-109.2019	-84.21468
F69L_S73I_T	-94.875	2.83856	47.26819	0	0.01	1	-106.8478	-82.90214
F69L_S73I_T	-100.875	2.96203	48.16249	0	0.01	1	-113.3686	-88.38134
F69L_S73I_T	-99.58929	2.83856	49.61692	0	0.01	1	-111.5621	-87.61643
F69L_S73I_T	-35.01786	2.83856	17.44644	0	0.01	1	-46.99071	-23.045
F69L_S73I_T	-105.5892	2.83856	52.60621	0	0.01	1	-117.5621	-93.61643
F69L_S73I_T	-98.875	2.96203	47.2076	0	0.01	1	-111.3686	-86.38134
F69L_S73I_T	-98.475	3.12671	44.5403	0	0.01	1	-111.6632	-85.28673
F69L_S73I_T	-92.675	3.12671	41.91695	0	0.01	1	-105.8632	-79.48673
F69L_S73I_T	-102.625	2.74231	52.92392	0	0.01	1	-114.1918	-91.05812
F69L_S73I_T	-26.875	2.74231	13.85949	0	0.01	1	-38.44188	-15.30812

Sig equals 1 indicates that the difference of the means is significant at the 0.01 level.
Sig equals 0 indicates that the difference of the means is not significant at the 0.01 level.

Comparison of HcKCR2 & HcKCR2_V73I (text)

Mann-Whitney Test (8/4/2022 14:43:06)

Notes

X-Function	Mann-Whitney Test
User Name	egovorunova
Time	8/4/2022 14:43:06
Data Filter	No

Input Data

	Data	Range
1st Data Range	[HcKCR2]Sheet1!HcKCR2	[1*:7*]
2nd Data Range	[HcKCR2]Sheet1!V73I	[1*:8*]

Descriptive Statistics

	N	Min	Q1	Median	Q3	Max
HcKCR2	7	-79.4	-77.4	-76.4	-66.4	-66.4
V73I	8	-83.4	-80.15	-78.9	-77.4	-74.4

Ranks

	N	Mean Rank	Sum Rank
HcKCR2	7	10.64286	74.5
V73I	8	5.6875	45.5

Test Statistics

	U	Z	Asymp. Prob>|U|
	46.5	2.10386	0.03539

Null Hypothesis: F(x) = G(y)

Alternative Hypothesis: F(x) <> G(y)

At the 0.05 level, the two distributions are significantly different.
