# Supplementary material for: Structural Foundations of Potassium Selectivity in Channelrhodopsins
Source: mBio. 2022 Nov 22;13(6):e03039-22. doi: 10.1128/mbio.03039-22 (PMC9765531; doi:10.1128/mbio.03039-22)
Supplement: TABLE S2 [file mbio.03039-22-s0002.docx]

| **Wavelength** | **Light power (mW mm^-2^)** |
| --- | --- |
| 400 | 5.93 |
| 410 | 6.40 |
| 420 | 6.63 |
| 430 | 6.82 |
| 440 | 7.02 |
| 450 | 7.43 |
| 460 | 8.05 |
| 470 | 8.50 |
| 480 | 8.25 |
| 490 | 7.90 |
| 500 | 7.61 |
| 510 | 7.38 |
| 520 | 7.27 |
| 530 | 7.19 |
| 540 | 7.05 |
| 550 | 6.95 |
| 560 | 6.81 |
| 570 | 6.74 |
| 580 | 6.58 |
| 590 | 6.48 |
| 600 | 6.34 |
| 610 | 6.13 |
| 620 | 5.81 |
| 630 | 5.55 |
| 640 | 5.37 |
| 650 | 5.22 |
